# Better safe than sorry?—On the influence of learned safety on pain perception

**DOI:** 10.1371/journal.pone.0289047

**Published:** 2023-11-07

**Authors:** Anna-Lena Zillig, Paul Pauli, Matthias Wieser, Philipp Reicherts

**Affiliations:** 1 Department of Psychology, University of Würzburg, Würzburg, Germany; 2 Department of Clinical Psychology, Erasmus University of Rotterdam, Rotterdam, Netherlands; 3 Department of Medical Psychology and Sociology, University of Augsburg, Augsburg, Germany; Liverpool John Moores University, UNITED KINGDOM

## Abstract

The experience of threat was found to result—mostly—in increased pain, however it is still unclear whether the exact opposite, namely the feeling of safety may lead to a reduction of pain. To test this hypothesis, we conducted two between-subject experiments (N = 94; N = 87), investigating whether learned safety relative to a neutral control condition can reduce pain, while threat should lead to increased pain compared to a neutral condition. Therefore, participants first underwent either threat or safety conditioning, before entering an identical test phase, where the previously conditioned threat or safety cue and a newly introduced visual cue were presented simultaneously with heat pain stimuli. Methodological changes were performed in experiment 2 to prevent safety extinction and to facilitate conditioning in the first place: We included additional verbal instructions, increased the maximum length of the ISI and raised CS-US contingency in the threat group from 50% to 75%. In addition to pain ratings and ratings of the visual cues (threat, safety, arousal, valence, and contingency), in both experiments, we collected heart rate and skin conductance. Analysis of the cue ratings during acquisition indicate successful threat and safety induction, however results of the test phase, when also heat pain was administered, demonstrate rapid safety extinction in both experiments. Results suggest rather small modulation of subjective and physiological pain responses following threat or safety cues relative to the neutral condition. However, exploratory analysis revealed reduced pain ratings in later trials of the experiment in the safety group compared to the threat group in both studies, suggesting different temporal dynamics for threat and safety learning and extinction, respectively.

**Perspective**: The present results demonstrate the challenge to maintain safety in the presence of acute pain and suggest more research on the interaction of affective learning mechanism and pain processing.

## Introduction

The modulation of pain perception by affective states is well established [1] such that negative emotions were found to increase pain [2–4], while positive affective states result in decreased pain [2, 4–12]. Especially threat, resulting from the anticipation of an aversive outcome (e.g., electrical shocks) was found to induce a negative affective state and alter the perception of concurrently administered pain, leading–mostly–to hyperalgesia [4, 13, 14]. Previously, we observed an increase in subjective and neurophysiological indices of pain following threat induction [2]. In this experiment–as is often the case when employing threat conditioning or related paradigms–the actual effect of a cue announcing potential danger (e.g., CS+) is inferred by direct comparison to a safe condition (e.g., CS-). However, such experimental approaches are not necessarily well-equipped to determine the net effect of safety, given that safety is dependent on the amount of threat being induced. Therefore, the question remains, whether safety, as the conceptual and motivational opposite of threat, might have an orthogonal–that is pain reducing–effect? Animal studies demonstrated that the induction of safety–for example by a negative association of an auditory conditioned stimulus (CS) with an aversive event (US)–was positively correlated with explorative behavior, which is indicative for positive affective states [15–18]. In line with the motivational priming hypothesis [19, 20], postulating two opposite motivational systems, where the *aversive* system is activated by potential or actual threat, in contrast to the *appetitive* system, which is activated by stimuli that predict survival or positive outcomes, safety should activate the appetitive system and thus reduce the perception of pain. So far, research on the experimental induction of safety–in humans–is scarce [21], let alone its impact on pain, although the modulation of pain by safety might hold great potential for our understanding and the management of pain by psychological factors.

In the present study, we employed the so called *explicit unpaired procedure* to induce safety, which originally derives from animal research [16, 22–24] and was already successfully adapted to human research [21]. According to the paradigm, learned safety results from a negative association through strict temporal separation of a neutral stimulus (NS) and an aversive unconditioned stimulus (US), resulting in a safety signal, (negatively conditioned stimulus, CS-) [25].

We conducted two experiments, closely following the design by Pollak, Rogan [21]. Each experiment included two groups of participants, who first completed either a threat or a safety conditioning procedure, followed by an identical test phase, where the previously conditioned threat vs. safety cue (CS) and a newly introduced neutral stimulus (NEW) were presented simultaneously with heat pain stimuli. In experiment 2, CS-US timing during acquisition was modified, since previous studies found that the interstimulus interval (ISI; interval between CS onset and US onset) is crucial when inducing threat and safety respectively [26, 27]. Therefore, we increased the maximum length of the ISI from 15-25s to 12-32s in the safety group. Thereby, potential trace conditioning should be prevented, which was found to occur even if a CS was followed by a US within an interval of up to 10s [28]. Secondly, we included additional verbal instructions before threat or safety acquisition in experiment 2, respectively. The safety group was informed that the presentation of the CS was a reliable indicator of safety, meaning that—for sure—no electrical shock would ever be administered during or directly following the CS. The threat group instead was informed that the CS was indicative for threat and would be followed in most of the cases by an electrical shock. Lastly, we raised CS-US contingency in the threat group from 50% to 75%, to facilitate threat conditioning and reduce ambiguity regarding the predictive value of the threat signal. In both experiments, we complemented pain reports and affective ratings of the visual cues with psychophysiological measure of pain and emotional responses (heart rate, HR; skin conductance, SC) [26, 29–31]. Based on the reviewed findings, we expected a reduced perception of pain following the presentation of the CS- compared to the NEW cue for the safety group, while in the threat group, pain following the CS+ should be increased compared to the NEW cue. Successful threat and safety induction should become evident by physiological responses and affective ratings during acquisition and test phase.

## Experiment 1

### Materials and methods

#### Participants

In total 94 (65 women) participants were recruited via the online platform SONA Systems (Sona Systems Ltd., Tallinn, Estonia) by the University of Würzburg and received 14€ for participation. Participants did not take psychopharmacological or pain medication within the last 24 hours and had no current or prior history of chronic pain (self-report). Further, inclusion criteria were age between 18 and 39 years, and no previous or current psychiatric diagnosis (self-report). Written informed consent was obtained from all participants for inclusion in the study. Four participants terminated the experiment early due to elevated discomfort, thus were excluded from final data analysis leaving a final sample size of 90 (61 women; age *M* = 24.69, *SD* = 3.99). Furthermore, participants with a mean pain rating of 0 on one or both dimensions were excluded from analysis of the pain ratings. This affected one participant in experiment 1. Optimal sample size was calculated a priori via G*Power, Version 3.1.9.2, University of Kiel, Germany [32]: assuming an effect size of 0.3, alpha error of .05 and power of > 0.80, recommended sample size was 90 for an ANOVA with fixed effects and two groups.

Participants were pseudo-randomly assigned to one of the two experimental groups: safety group (*n* = 46, 33 females) or threat group (*n* = 44, 28 females). Participants of the safety group (*M* = 23.63 *SD* = 3.65) were slightly younger than the ones of the threat group (*M* = 25.80, *SD* = 4.06; *t*(88) = 2.66; *p* = .01), (Table 1). Therefore, we checked for a potential linear association between pain and affective cue ratings during the test phase and age and found no significant correlation. Additionally, performed ANCOVAs controlling for age, revealed no significant effect on the pain ratings. Participants completed several questionnaires before and after the main experiment. More detailed information on the questionnaires can be found in the supplement (see S1 Table).

**Table 1:** Mean scores of pain threshold in the two experimental groups.

*Measure*	*safety group (n = 46)*		*threat group (n = 44)*		t	P
	M	SD	M	SD		
Age	23.63	3.65	25.66	3.98	2.52	.01*
Heat pain threshold (°C)	43.11	1.66	43.01	2.56	-.22	.83
Administered heat pain (°C)	45.11	1.66	45.00	2.54	-.24	.81
Electric pain threshold (mA)	0.75	0.54	0.87	0.58	1.05	.29
Administered electric pain (°C)	1.53	1.08	1.79	1.16	1.12	.26

#### Thermal pain stimulation

The thermal pain stimuli were delivered via a thermal stimulator and a thermode with an active area of 25×50 mm (Somedic SenseLab AB, Sösdala, Sweden). The thermode was attached to the volar forearm of the left arm. The individual pain threshold was assessed prior to the actual experiment using the *method of adjustment* [33, 34]. For that, participants were first familiarized with the thermode before they adjusted the thermode’s temperature starting from a baseline temperature of 35°C by pressing 2 buttons of the keypad (± 0.5°C/keystroke with a maximum temperature of 49°C) until they reached a level of thermal sensation that they perceived as just being painful. This procedure was repeated three times and the average of all three temperatures was used as the final pain threshold (PT). The average pain threshold temperature was *M* = 43.09°C, *SD* = 2.16, and did not differ between groups (see Table 1). During the experiment, we used the individual PT plus 2°C as target temperature (TT) to achieve a moderately painful stimulation [33, 34]. A practice pain stimulation was presented right before the beginning of the acquisition and right before the test phase to make sure that the selected temperature was still rated as moderately painful and to reduce arousal and ambiguity due to anticipation of the heat pain stimulation. Heat pain stimuli were applied starting at a baseline temperature of 10°C below TT and rose at a rate of 5°C/s. TT was presented for 5s and afterwards the thermode cooled down to baseline temperature and remained there until the next trial. To prevent sensitization to the pain stimulus, the position of the thermode was changed from the proximal to the distal part of the volar forearm or vice versa (position order was counterbalanced across participants), [35, 36] after the first half of the test phase (i.e. after 8 pain trials).

As the design of the present study was inspired by previous studies on the influence of threat on pain [2, 3], we aimed at a clear distinction between threat induction on the one hand and pain induction on the other hand and therefore used aversive electrical stimuli to induce a feeling of threat, whereas heat pain was used to assess pain later on.

#### Electrical stimulation

Electrical stimulation served as unconditioned stimulus (US), as it usually is very brief and abrupt and mostly perceived as quite aversive and thus was found to reliably elicit conditioned threat responses [26]. Electrical stimulation was delivered via surface bar electrodes consisting of two gold-plated steel disks (9 mm diameter, 30 mm spacing) attached to the right calf. The electrical stimuli lasted for 100ms each and were generated by a constant-current stimulator (Digitimer DS7A, Digitimer Ltd., Welwyn Garden City, UK). Prior to the main experiment, the individual threshold was assessed. The procedure consisted of two ascending and 2 descending series of stimulations, starting from 0 mA with increasing stimulus intensity in steps of 0.5 mA steps [7]. Participants had to rate verbally how painful they experienced each stimulation on a 10-point NRS ranging from 0–10 (from “no pain at all” up to “unbearable pain”). Intensities, which were perceived as just being painful were averaged and served as pain threshold. During the experiment, electrical stimuli were calibrated to the individual PT (*M* = 0.81, *SD* = 0.69 mA) plus 100% (max 10 mA) of the intensity to achieve a moderately painful, sufficiently aversive stimulation and prevent habituation. Right before the beginning of the main part of the experiment, one electrical stimulus was administered to ensure that the US was experienced as aversive. In case participants rated the US below 5 on the NRS, the stimulus workup procedure was repeated.

For the threat group, US were presented with visual cue (NS) offset at a contingency of 50% to establish a threat cue (CS+). For the safety group US and visual cue were presented strictly separate in time from each other (ISI min. 15s.) to establish a safety signal (CS-).

### Measures

#### Heat pain ratings

Before the experiment started, the distinction of pain intensity and pain unpleasantness was explained to the participants [37]. Participants rated heat pain stimuli using a digitized visual analogue scale (VAS), ranging from 0 = not painful at all / not unpleasant at all to 100 = extremely painful/ extremely unpleasant.

#### Heart rate

For the recording of the electrocardiography (ECG) three electrodes were attached on the torso of the participant: on the right collarbone, the left lower costal arch, and the ground electrode on the left lower side of the torso (3-channel derivation of Nehb; ASCII coding). The raw ECG-signal was filtered with a 30 Hz high-pass filter. Using the Vision Analyzer software (BrainProducts, Munich, Germany), R-waves were detected and manually inspected for artefacts, afterwards the inter-beat-intervals were calculated and converted into continuous heart rate [38]. Data was segmented into time intervals of five seconds before until 20s after cue onset. To evaluate cue and pain responses during the test phase, the HR signal was baseline corrected relative to a 5 second interval before the visual cue onset. Twenty time-bins were calculated by averaging intervals of 1 second. Wide time intervals were analyzed to capture potentially delayed onsets of psychophysiological reactions following heat pain stimulation [39]. Data of one participant was excluded from HR analysis due to recording failure.

#### Skin conductance

For the recording of skin conductance (SC) two 22/10mm Ag/AgCl surface electrodes (electrode gel: 0.5% NaCl) were attached to the thenar and hypothenar of the left hand. Skin conductance was continuously recorded at a sampling rate of 20 Hz. using a Brain Vision Recorder and V-Amp amplifier (Brain Products, Munich, Germany). Data was averaged across all trials per condition (CS/ CS.NEW), baseline corrected relative to a five second interval before visual cue onset, and twenty 1 second time-bins following cue onset were calculated for further analysis.

#### Cue ratings

In the beginning and twice during the acquisition and test phase (i.e., 5 times), ratings of the different visual cues were captured. Participants rated on a 9-point scale how threatened or safe they felt in presence of the cue (1 = not at all; 9 = very much). Furthermore, they rated valence and arousal during presentation of the visual cues, using the 9-point Self-Assessment-Manikin (SAM) [40], ranging from 1 = very unpleasant to 9 = very pleasant and 1 = not at all arousing to 9 = very arousing, respectively. Last, participants indicated on a 100-step VAS how much they expected an electrical stimulation following each visual cue (contingency; 0 = not at all likely to 100 = very likely).

The preacquisition ratings at the beginning of the experiment did not indicate differences between CS and CS.NEW, neither for the safety nor for the threat group (Table 2) in experiment 1.

**Table 2:** Results of the paired t-tests for the comparison of the preacquisition stimulus ratings of CS and CS.NEW for both groups.

*Measure*	safety group					threat group				
		*M*	*SD*	*t*	*p*		*M*	*SD*	*t*	*p*
threat rating	CS	2.30	1.66	-.08	.93	CS	2.39	1.82	.16	.55
	CS.NEW	2.33	1.81			CS.NEW	2.14	1.88		
safety rating	CS	6.61	2.19	.00	.99	CS	6.77	1.93	-.08	.94
	CS.NEW	6.61	2.17			CS.NEW	6.80	1.84		
valence rating	CS	6.28	1.28	.74	.46	CS	6.30	1.41	-1.20	.24
	CS.NEW	6.11	1.64			CS.NEW	6.52	1.39		
arousal rating	CS	3.54	2.06	.37	.71	CS	3.18	1.66	-.46	.65
	CS.NEW	3.43	1.9			CS.NEW	3.30	1.61		
contingency	CS	18.67	25.05	-.35	.97	CS	22.55	27.51	-.03	.97
	CS.NEW	20.33	23.88			CS.NEW	22.68	27.96		

#### Procedure

**Fig 1:**
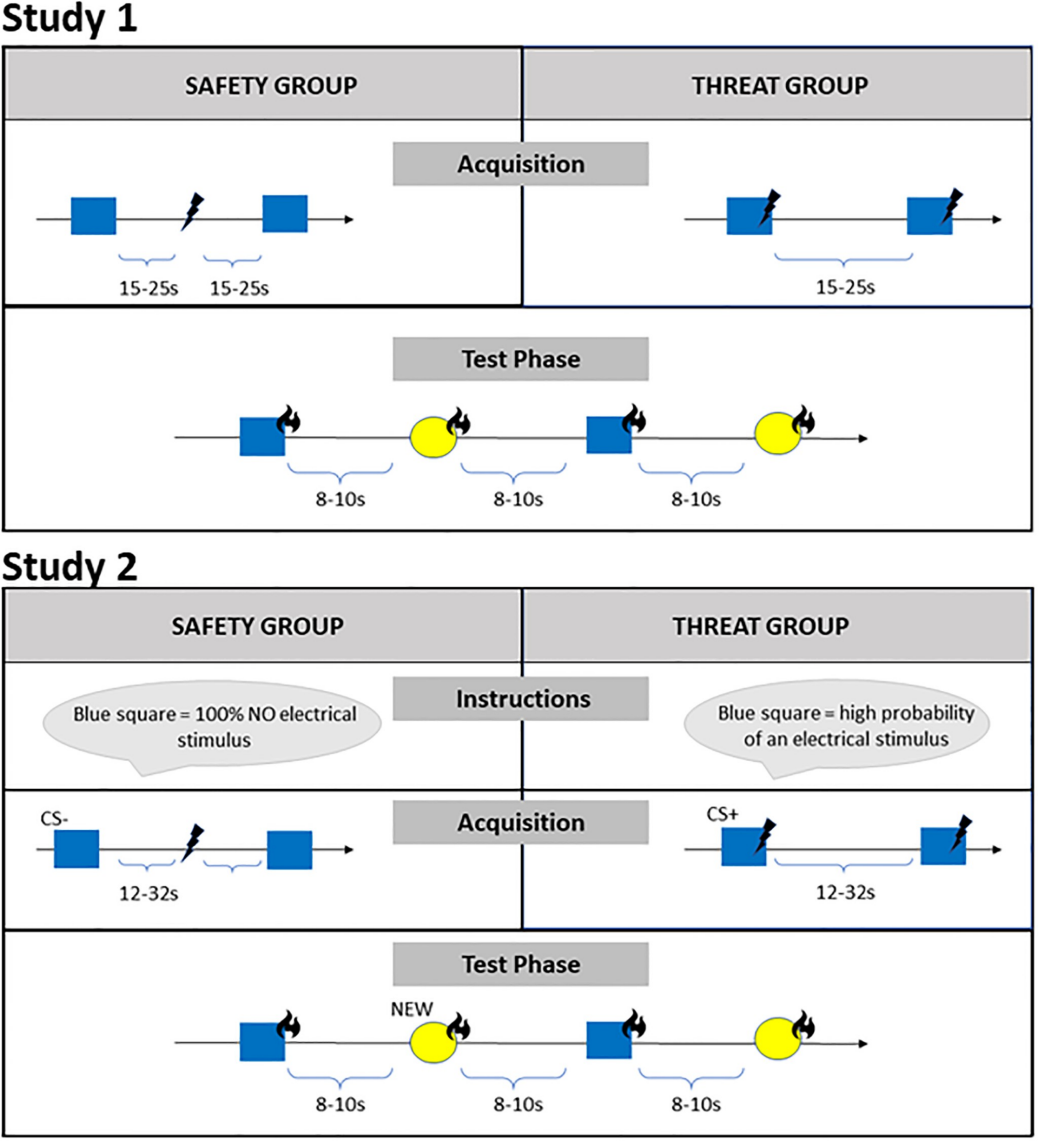
Experimental design of acquisition and test phase for both groups of experiment 1 and 2. Shown are the CS (blue square) during acquisition, associated with safety in one group (strict temporal separation of aversive and neutral stimulus: NS // US → CS-) and with threat in the other (temporal association of the stimuli: NS + US → CS+). The following test phase was identical for both groups and included the CS as well as a new visual cue (yellow circle, NEW) simultaneously presented with heat pain stimuli.

To investigate the influence of threat vs. safety on heat pain processing, respectively, participants first underwent a safety or threat induction (conditioning procedure using an aversive electrical shock as US) before entering a test phase during which the modulation of heat pain stimuli by safety or threat was investigated (for an overview of the design see Fig 1).

In detail, participants were pseudo-randomly allocated to either the safety or the threat group. Arriving at the laboratory, participants received written information about the study procedure and signed informed consent. First, participants filled out questionnaires (see S1 Table), afterwards the electrodes for ECG, SC measures and electrical stimulation were attached, as well as the thermode.

Participants were seated in front of a computer screen. Instructions, rating scales and stimuli were presented via the software Presentation^®^ (Version 20.0 Neurobehavioral Systems Inc., Albany, CA, USA). Then, the individual heat and electrical pain threshold was assessed. Afterwards the use of the rating scales for valence, arousal, contingency, threat, and safety was instructed. Afterwards the two visual cues (NS, see Fig 1) were presented and rated. In the following experiment one cue served either as threat (CS+) or safety cue (CS-), dependent on the experimental group, while the other served as neutral reference during the test phase (NEW). The blue square (RGB: 0, 0, 255) had the dimensions 500 x 500 pt. And the yellow circle (RGB: 255, 255, 0) had a diameter of 564 pt. Then, participants practiced the use of the VAS for the pain ratings and received instruction regarding the distinction of pain intensity and unpleasantness. Immediately preceding the main experiment, one heat pain stimulus and one electrical stimulus was administered and rated by the participant. The implementation of safety and threat was conceptualized via a conditioning procedure, i.e., the acquisition phase, which was followed by the test phase (see Fig 1 for an illustration of the design).

*Acquisition*: The manipulation varied according to the experimental group (safety vs. threat). Each trial started with a central fixation cross presented on a grey background (RGB: 200, 200, 200). After 15 to 25 seconds (randomized), the visual cue, either a blue square or a yellow circle (counterbalanced across all participants), was presented in the middle of the screen. The cue remained on the screen for 10 seconds before disappearing (cue offset). In the threat group, the electrical stimulus (US) was presented with cue offset (contingency: 50%), establishing a threat cue. In the safety group, the electrical stimulus and visual cue were presented separately in time (interstimulus interval varying from 15-25s), in accordance with the so called explicit unpaired procedure, adapted from Pollak, Rogan [21] in order to make participants learn about the negative association of cue and US. The acquisition consisted of 2 blocks á 8 trials. After each block cue ratings were gathered.

*Test phase*: The test phase was identical for the two groups. Again, each trial started with a central fixation cross, followed by the presentation of the previously established threat or safety cue or a newly introduced visual cue (NEW, either a circle or a square) simultaneously with the administration of heat pain stimuli onto the participant’s forearm. After each trial, participants rated the heat pain stimuli regarding pain intensity and unpleasantness. The inter-trial interval was set to 8-10s (randomized). The test phase consisted of 2 blocks á 4 trials per condition plus 1 booster trial in each block. To prevent rapid extinction, during booster trials an electrical stimulus was presented—analogue to the acquisition phase—either at CS offset (threat group) or during ITI (safety group). Booster trials were presented each after half of the block (4^th^ trial). Booster trials were excluded from the statistical analyses. Again, following each block cue ratings were collected. The study was approved by the local ethic committees of the Psychological Institute of the University of Würzburg.

#### Statistical analysis

Data was analyzed using IBM SPSS statistics software version 25 (IBM Corp., Armonk, NY, USA). Pain ratings (intensity and unpleasantness) were analyzed separately by repeated-measures ANOVAs including the between-subjects factor *group* (safety vs. threat) and the within-subjects factors *cue* (CS vs NEW) and *time* (trials 1–8 in the test phase).

Cue ratings of the test phase were also analyzed with repeated-measures ANOVAs including the within-subjects factor *cue* and the between-subjects factor *group*. Additionally, we analyzed cue ratings of the CS+ or CS- of the acquisition *and* test phase (4 ratings in total) to explore conditioning and extinction processes throughout the time course of the experiment, applying the within-subjects factor *trial* (4 levels) and the between-subjects factor *group*.

For analysis of HR and SC during the test phase, we used a repeated-measures ANOVA including the between-subjects *group* (safety vs. threat) and the within-subjects factor *cue* (CS vs NEW), and the within-subjects factor *time* (twenty 1-second bins).

Significance level was defined as *P* < 0.05 and report partial eta-squared *η*_*p*_^*2*^ is reported as measure of effect size. In case assumption of sphericity was violated (Mauchly), the Greenhouse-Geisser correction was applied. Significant main effects and interactions were followed up by subsequent one-way ANOVAs, simple contrasts, and t-tests, respectively. In case of exploratory analyses, post-hoc analyses were Bonferroni corrected and corrected *p*-values are reported.

### Results

#### Pain ratings

**Fig 2:**
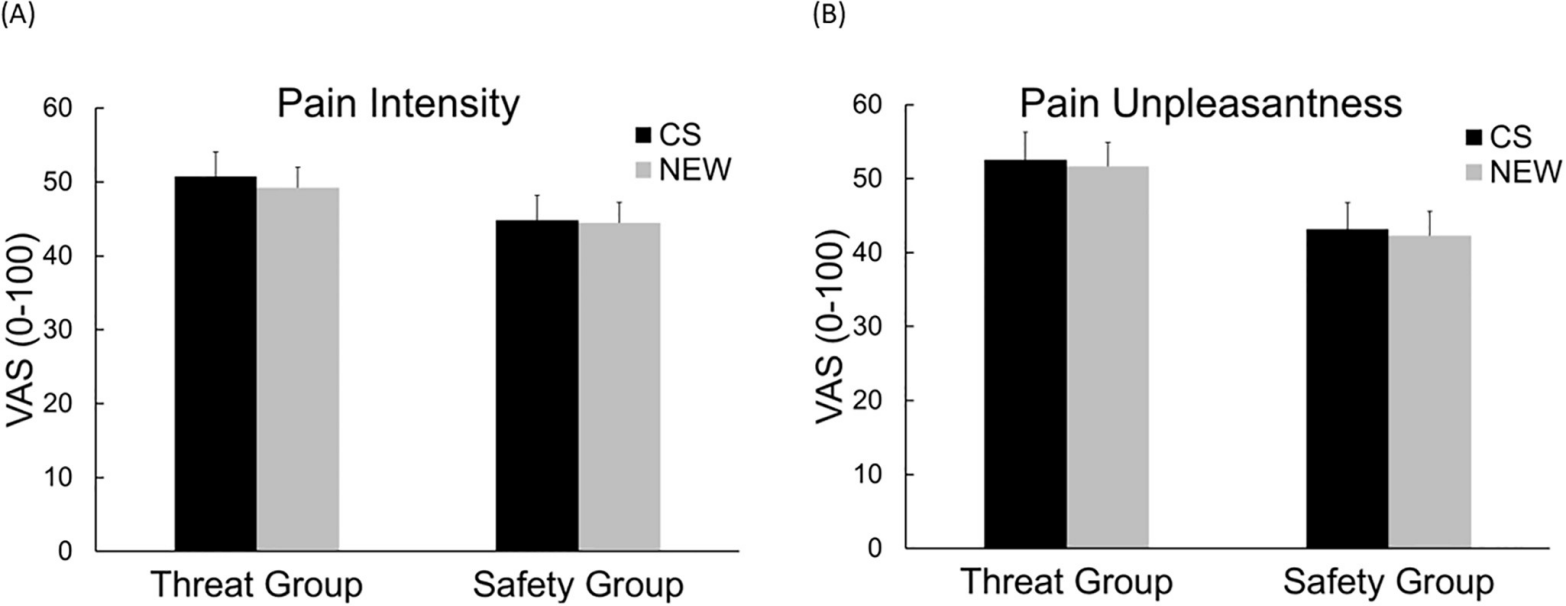
Mean pain intensity and unpleasantness ratings. Mean (+SEM) (A) Pain Intensity Ratings and (B) Pain Unpleasantness Ratings of the Test Phase for both groups, separately for safety / threat (CS) and new trials (NEW), respectively.

**Fig 3:**
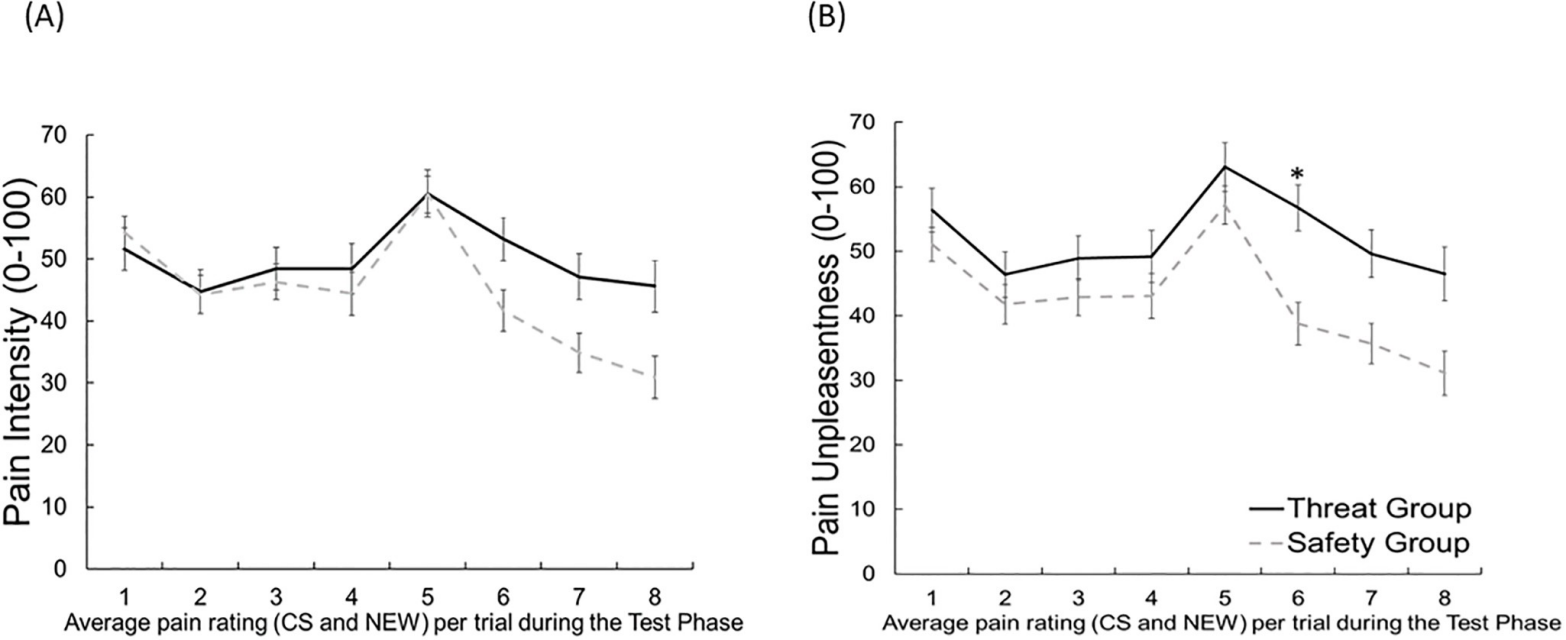
Mean pain intensity ratings and pain unpleasantness ratings, averaged across cues, separately for both groups. Mean (+SEM) (A) Pain Intensity Ratings and (B) Pain Unpleasantness Ratings, averaged across cues, separately for both groups. Following the fourth trial, the thermode was relocated and stimulation continued on a different patch. * *p* < .05.

Analysis of *pain intensity* revealed neither a significant main effect of *group*, *F*(1, 87) = 1.64, *p* = .20, *ηp*^2^ = .02, nor of *cue*, *F*(1, 87) = 1.05, *p* = .31, *ηp*^2^ = .01, nor a significant interaction of *cue* and *group*, *F*(1, 87) = 0.42, *p* = .52, *ηp*^2^ = .01 (Fig 2). However, we found a significant interaction of *time* and *group*, *F*(7, 609) = 6.34, *p <* .001, *ηp*^*2*^ = .07, which results from higher pain ratings in the threat compared to the safety group, especially at the end of the test phase: *Exploratory analysis* for the second half of the test phase revealed for trials 6 (mean difference: 11.58, *t*(87) = 2.38, *p* = .02), 7 (mean difference: 12.24, *t*(87) = 2.53, *p* = .01) and 8 (mean difference: 14.74, *t*(87) = 2.73, *p* = .01) higher pain ratings for the threat group compared to the safety group irrespective of the presented cue (CS+/- or NEW), however, this effect became non-significant after accounting for multiple testing (Bonferroni corrected *p*-value = 0.0125 (Fig 3).

To get a better understanding of the temporal dynamics, we exploratory analyzed only the first trial. However, this did not reveal any effect for pain intensity.

For *pain unpleasantness* ratings there was no main effect of *cue*, *F*(1, 87) = .79, *p* = .38, *ηp*^*2*^ = .01, nor a significant interaction of *cue* and *group*, *F*(1, 87) < 0.01, *p* = .99, *ηp*^2^ = .01.

We found a main effect of *group*, *F*(1, 87) = 3.88, *p* = .05, *ηp*^*2*^ = .04 due to lower ratings of the safety group (*M* = 42.71, *SD* = 23.65) compared to the threat group (*M* = 52.11, *SD* = 21.38), (Fig 2). This main effect was further qualified by a significant interaction of *time* and *group*, *F*(7, 609) = 3.30, *p* = .002, *ηp*^2^ = .04.

*Exploratory analysis* indicates, similar to the pain intensity ratings, that this is likely due to higher ratings of the threat compared to the safety group at the end of the test phase: pain ratings in trials 6 (mean difference: 17.96, *t*(87) = 3.41, *p* < .001), were significantly different from each other. However, for trial 7 (mean difference: 13.95, *t*(87) = 2.54, *p* = .01), and 8 (mean difference: 15.39, *t*(87) = 2.60, *p* = .01), this trend became non-significant after accounting for the Bonferroni correction based on eight tests and a corresponding p-value of *p* = 0.00625.(Fig 3). To get a better understanding of the temporal dynamics, we exploratory analyzed only the first trial. However, this did not reveal any effect for pain unpleasantness.

#### Heart rate

**Fig 4:**
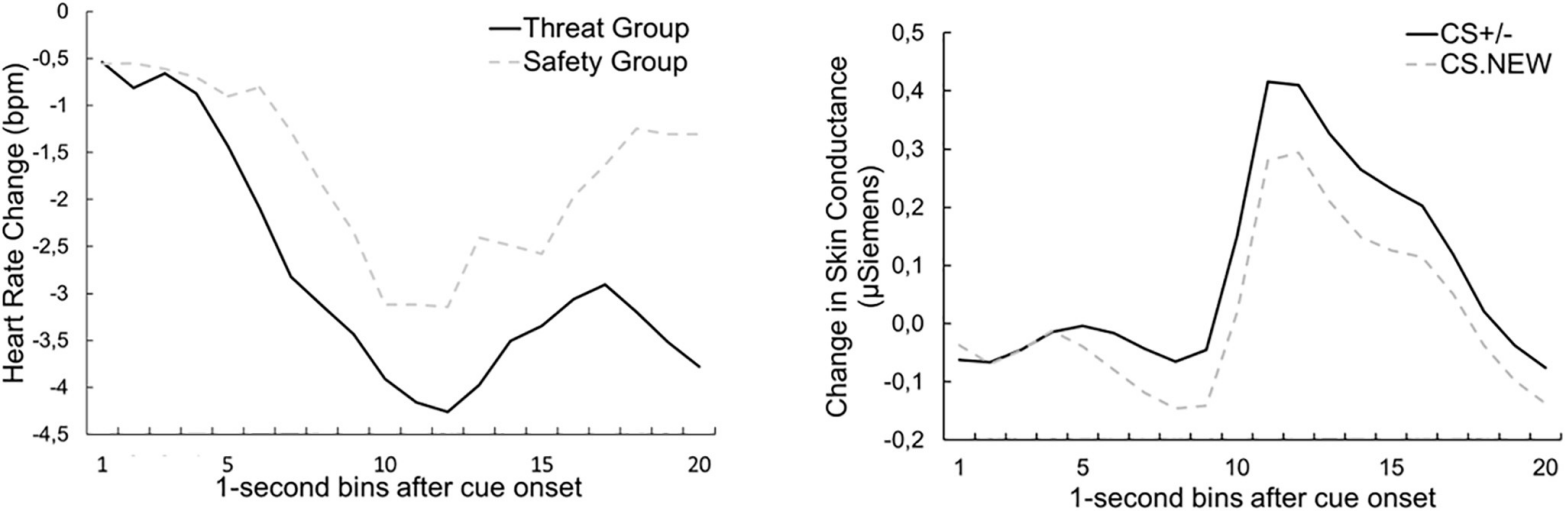
Time course of heart rate and of the change in skin conductance. (A)Time course of Heart Rate (1s time bins, baseline-corrected 5 s before cue onset), averaged across both CS types, separately for both experimental groups, during the Test Phase and (B) Mean time course (1-s bins) of the change in Skin Conductance (baseline-corrected 5 s before cue onset) during the Test Phase for the two cue types.

Analysis of heart rate revealed a significant main effect of *time*, *F*(19, 1672) = 23.59, *p* < .001, *ηp*^*2*^ = .21, ε = .19, due to a cue (deceleration) and pain response (acceleration), respectively. However, this effect was independent from the type of cue being presented or the experimental group (interaction of *cue* and *group*, *F*(1, 88) = 0.71, *p* = .40, *ηp*^2^ = .01). There was a trend for the (interaction of *time* and *group*: *F*(19, 1672) = 1.47, *p* = .09, *ηp*^2^ = .02, likely indicating smaller pain related HR responses in the safety group irrespective of the presented cue. There were no further significant main effects or interactions (main effect of *cue*: *F*(1, 88) = .05, *p* = .83, *ηp*^2^ < .01, interaction of *time* and *cue F*(19, 1672) = .97, *p* = .44, *ηp*^2^ = .01, *ε* = .28, interaction of *time*, *cue* and *group*: *F*(19, 1672) = .50, *p* = .79, *ηp*^2^ = .01). The mean time course for both groups is shown in Fig 4.

#### Skin conductance

Analysis of skin conductance revealed a significant main effect of *time* during the test phase, *F*(19, 1672) = 23.53, *p* < .001, *ηp*^*2*^ = .21, ε = .08, indicating a SC reaction following the heat pain stimulus (Fig 4). The CS(+/-) led to higher skin conductance changes than the NEW cue, regardless of the experimental group (main effect *cue*: *F*(1, 88) = 5,78, *p* = .01, *np*^*2*^ = .06), see Fig 4. These main effects were further qualified by a significant interaction of *cue* and *time*, *F*(19, 1672) = 2.93, *p* = .044, *ηp*^*2*^ = .03, ε = 13, from seconds 6 until seconds 15, SC was significantly higher for the conditioned CS (+/-) compared to the NEW cue (all *p*s < .05, uncorrected). There was no significant interaction of *cue* and *group*, *F*(1, 88) = 0.57, *p* = .45, *ηp*^2^ = .01, nor of *cue*, *group*, and *time F*(19, 1672) = 0.54, *p* = .95, *ηp*^*2*^ = .01.

#### Cue ratings

*Cue ratings during the test phase*. Analysis of *threat rating* during the test phase revealed a significant main effect of *cue*, *F*(1, 88) = 18.69, *p* < .001, *ηp*^*2*^ = .18, resulting from higher threat ratings for the CS compared to the NEW cue. There was neither a significant main effect of *group F*(1, 88) = 1.35, *p* = .25, *ηp*^*2*^ = .02, nor a significant interaction of *cue* and *group*, *F*(1, 88) = 2.31, *p* = .14, *ηp*^*2*^ *=* .03.

Analysis of *safety rating* revealed a significant main effect of *cue*, *F*(1, 88) = 16.76, *p* < .001, *ηp*^*2*^ = .16, resulting from higher safety ratings for the NEW compared to the CS, which was further qualified by a significant interaction of *cue* and *group*, *F*(1, 88) = 5.53, *p* = .02, *ηp*^*2*^ = .06. Separate analysis for both groups revealed significantly higher safety ratings for the NEW compared to the CS cue in the threat group, *F*(1, 43) = 19.25, *p* < .001, *ηp*^*2*^ = .31, while the same comparison was not significant in the safety group, *F*(1, 45) = 1.64, *p* = .21, *ηp*^*2*^ = .04. There was no significant main effect of *group*, *F*(1, 88) = .67, *p* = .80, *ηp*^2^ = .001.

*Arousal ratings* revealed a significant main effect of *cue*, *F*(1, 88) = 10.88, *p* < .001, *ηp*^*2*^ = .11, resulting from higher arousal ratings for the CS compared to the NEW cue. There was no significant interaction of *cue* and *group*, *F*(1, 88) = 0.33, *p* = .57, *ηp*^*2*^ = .004, as well as no significant main effect of *group*, *F*(1, 88) = .43, *p* = .51, *ηp*^2^ = .005.

Analysis of *valence ratings* revealed a significant main effect of *cue*, *F*(1, 88) = 14.16, *p* < .001, *ηp*^*2*^ = .14, which was qualified by a significant interaction of *cue* and *group*, *F*(1, 88) = 3.94, *p* = .05, *ηp*^*2*^ = .043. Separate analysis per group revealed a significant main effect of *cue* in the threat group only, here the NEW cue was rated more positive than the CS (*F*(1, 43) = 15.02, *p* < .001, *ηp*^*2*^ = .26), which was not the case for the safety group (*F*(1, 45) = 1.74, *p* = .19, *ηp*^*2*^ = .04). There was no significant main effect of *group*, *F*(1, 88) = 1.15, *p* = .29, *ηp*^2^ = .01.

**Fig 5:**
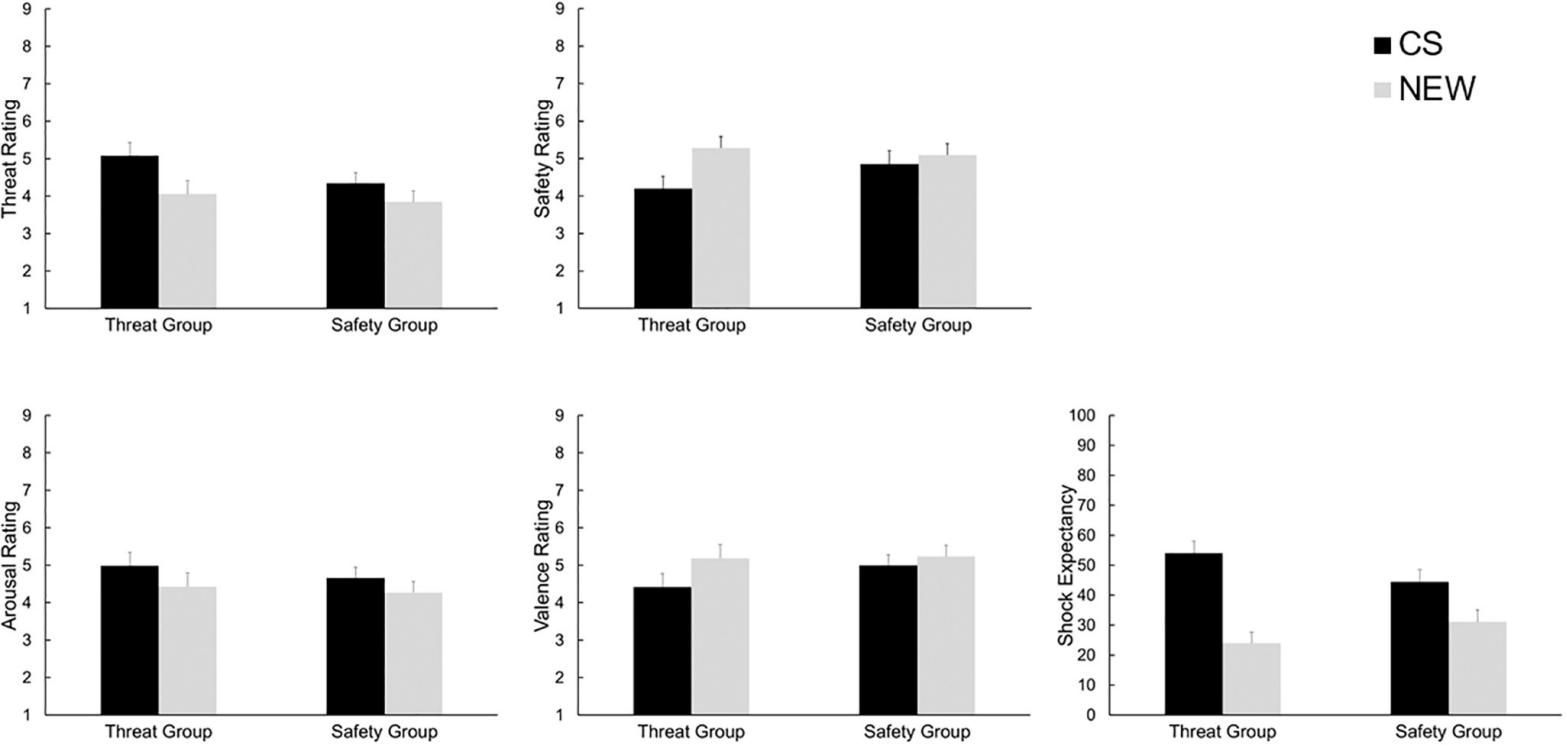
Cue ratings during the test phase. Mean (+ SEM) ratings for threat, safety, valence, arousal, and shock expectancy are depicted separately for both groups and cues, * *p* < .05.

Analysis of *shock expectancy* during the test phase revealed a significant main effect of *cue*, *F*(1, 88) = 71.84, *p* < .001, *ηp*^*2*^ = .45, resulting from higher shock expectancy ratings for the CS compared to the NEW cue. This main effect was further qualified by a significant interaction of *cue* and *group*, *F*(1, 88) = 10.63, *p* = .002, *ηp*^2^ = .011. Separate analysis for both groups revealed significantly higher expectancy ratings for the CS compared to the NEW cue in the threat group, *F*(1, 43) = 51.83, *p* < .001, *ηp*^*2*^ = .55, and in the safety group, *F*(1, 45) = 18.98, *p* < .001, *ηp*^*2*^ = .30, as well. Descriptively, the difference between the ratings of CS and NEW cue were bigger in the threat group (*M* = 30.01, *SD* = 3.97) compared to the safety group (*M* = 13.39, SD = 3.63). There was no significant main effect of *group*, *F*(1, 88) = .06, *p* = .81, *ηp*^2^ < .001. Cue ratings of the test phase are presented in Fig 5.

*Exploratory analysis*: *Cue ratings of the CS(+/-) across acquisition and test phase*

To map the time course of learning and extinction processes respectively, we additionally analyzed the cue ratings of the CS(+/-) during acquisition and test phase. The analysis demonstrates relatively stable ratings of the threat group across the experimental phases, while analysis of the ratings of the safety group suggests rapid safety extinction. Detailed analyses of the comparison of acquisition and test phase for the threat, safety, valence, arousal and shock expectancy ratings are presented in the supplement (S1 Fig).

## Experiment 2

Cue ratings during acquisition of experiments 1 suggest that the induction of safety takes longer compared to the induction of threat or valence and arousal ratings. Result of the test phase especially suggest that the safety induction led to rather unstable effects. Therefore, we decided to perform a series of methodological changes in experiment 2 to support the induction of safety in the first place. Even though during acquisition, the safety cue (CS-) and US (electrical shock) presentation were strictly separated in time, still participants might have positively associated cue and shock, as reflected in the US expectancy ratings, thus hampering the establishment of a robust safety signal. Accordingly, methodological changes were performed in experiment 2, to further support safety induction and prevent from early threat and safety extinction. In line with findings demonstrating the capacity of explicit instructions to induce threat that persists over prolonged intervals—even multiple experimental sessions [27]—and the notion that contingency awareness is especially crucial for the induction of safety [41], we included *additional* verbal instructions before threat or safety acquisition, respectively. The safety group was informed that the presentation of CS was a reliable indicator of safety, meaning that—for sure—no electrical shock would ever be administered during or directly following the CS. The threat group instead was informed that the CS was indicative for threat and would be followed in most of the cases by an electrical shock.

Furthermore, we increased the maximum length of the ISI between CS and US administration, now ranging from 12 to 32 seconds. Thereby, potential trace conditioning should be prevented, which was found to occur even if a CS was followed by a US within an interval of up to 10s [28]. Lastly, we raised CS-US contingency in the threat group from 50% to 75%, to facilitate threat conditioning and reduce ambiguity regarding the predictive value of the threat signal.

### Material and methods

#### Participants

In total 87 (63 women) participants (Table 3) were recruited via the online platform SONA Systems (Sona Systems Ltd., Tallinn, Estonia) by the University of Würzburg and received 14€ for participation. Exclusion criteria were the same as in experiment 1, additionally participation in experiment 1 was forbidden. Written informed consent was obtained from all participants for inclusion in the study. From originally 87 participants seven had to be excluded from the final analysis, resulting in a sample size of 80 (57 women; age *M* = 24.72, *SD* = 4.38). Five participants were excluded due to technical problems. Two participants reported somatic and psychological symptoms respectively and thus were excluded from the data set before further analyses. The participants were pseudo-randomly assigned to one of the two experimental groups: safety group (*n* = 39, 27 females) or threat group (*n* = 41, 30 females). Before the main experiment participants completed questionnaires as in experiment 1 (see S2 Table).

**Table 3:** Mean scores in pain threshold in the two experimental groups.

*Measure*	*safety group (n = 39)*		*threat group (n = 41)*		t	p
	M	SD	M	SD		
Age	25.08	5.03	24.39	3.68	-0.69	.49
Heat pain threshold (°C)	42.74	2.20	43.21	2.23	0.93	.35
Administered heat pain (°C)	44.72	2.17	45.18	2.01	0.99	.32
Electrical pain threshold (mA)	1.05	0.69	1.02	0.87	-0.19	.85
Administered electrical pain (°C)	2.11	1.38	2.04	1.66	-0.22	.83

#### Thermal pain stimulation

Thermal pain stimulation and assessment of the threshold was the same as in experiment 1. In case a thermal stimulation was not executed due to technical failure (in total in 17 trials), the missing rating was replaced by the mean of the condition. The average pain threshold temperature was *M* = 42.98°C, *SD* = 2.17 and did not differ between groups (Table 3).

#### Electrical stimulation

Electrical stimulation and assessment of the threshold was the same as in experiment 1. The average threshold was *M* = 1.04, *SD* = 0.88 mA. The only difference to experiment 1 was that the reinforcement rate for the threat group was raised from 50 to 75% during acquisition.

#### Procedure

The procedure of experiment 2 was very similar to experiment 1, thus the following description lists only deviations from experiment 1. After the baseline measurement of the CS on all dimensions we included *verbal instructions* about the properties of the cue in experiment 2: Depending on group assignment, different information about the threat vs. safety cue CS were given. The safety group was instructed that the CS would never be followed by an electrical stimulus, while the threat group was told that the CS would be followed by the electrical stimulus with high probability. Afterwards the visual cue was rated on all dimensions once again to see if the information was understood. Then, acquisition and test phase followed. The only difference to the procedure of experiment 1 during acquisition was an extended variance of the ISI in the safety group from 15-25s to now 12-32s to avoid a vague association of the CS- and US in the safety group. For the threat group the contingency of CS+ and US was increased from 50 to 75% (= 6 out of 8 trials per block) with the aim to increase threat association even further. The test phase did not differ from experiment 1. The design is shown in Fig 1.

### Measures and statistical analysis

In experiment 2 the same measures were assessed as described in experiment 1. Also, data analysis was analogue to experiment 1. Due to technical failure, heart rate could only be analyzed from 59 participants (28 in the threat group and 31 in the safety group), and skin conductance data was available only from 61 participants (28 in the threat group and 33 in the safety group).

The preacquisition ratings at the beginning of the experiment showed a difference of CS and CS.NEW for the safety ratings in both groups, with higher ratings in the threat group. The other measures revealed no significant differences in experiment 2 (Table 4).

**Table 4:** Results of the paired t-tests for the comparison of the preacquisition stimulus ratings of CS and CS.NEW for both groups.

*Measure*	*safety group*					*threat group*				
		*M*	*SD*	*t*	*p*		*M*	*SD*	*t*	*p*
threat rating	CS	2.46	2.10	1.05.08	.30	CS	3.80	2.57	.00	1.00
	CS.NEW	2.08	1.63			CS.NEW	3.80	2.57		
safety rating	CS	6.46	2.19	-2.62	**.01**	CS	6.00	2.54	-2.04	**.05**
	CS.NEW	7.10	1.86			CS.NEW	6.76	1.87		
valence rating	CS	6.05	1.55	-1.08	.29	CS	5.85	1.59	-1.99	.05
	CS.NEW	6.38	1.62			CS.NEW	6.41	1.41		
arousal rating	CS	3.62	1.73	.00	.99	CS	3.29	1.65	.39	.70
	CS.NEW	3.62	1.87			CS.NEW	3.17	1.86		
contingency	CS	22.72	24.94	.29	.77	CS	14.00	24.24	-.06	.96
	CS.NEW	21.51	24.22			CS.NEW	14.24	24.22		

### Results

#### Pain ratings

**Fig 6:**
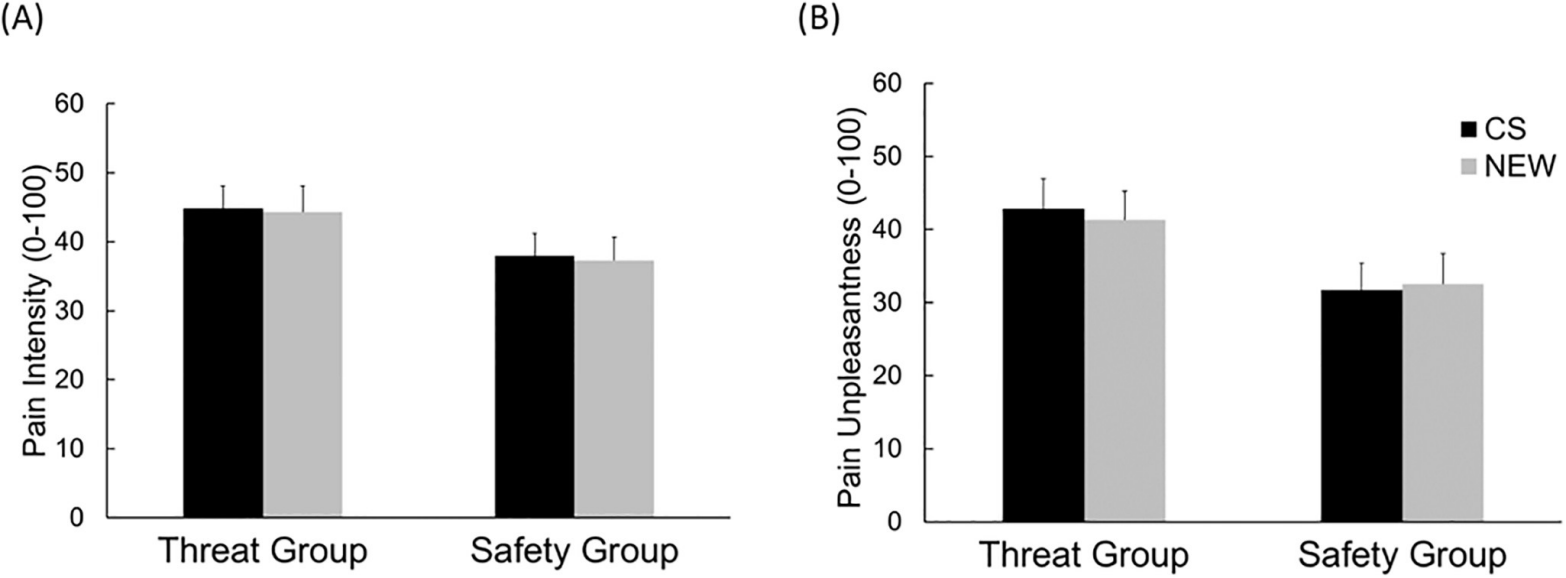
Pain intensity rating and pain unpleasantness ratings for both groups. Mean (+SEM) (A) Pain Intensity Rating and (B) Pain Unpleasantness Ratings for both groups, separately for safety / threat (CS) and new trials (NEW), respectively.

For *pain intensity* there was no significant main effect of *group*, *F*(1, 78) = 1.94, *p =* .17, *ηp*^*2*^ = .02, nor of *cue*, *F*(1, 78) = 0.55, *p* = .46, *ηp*^2^ = .01. Analysis revealed no significant interaction of *cue* and *group n*either, *F*(1, 78) = 0.01, *p* = .93, *ηp*^*2*^ < .001. The mean pain intensity and unpleasantness ratings are shown in Fig 6. Pain ratings differed over time for both groups, shown in the significant interaction of *time* and *group*, *F*(7, 546) = 2.37, *p* = .02, *ηp*^*2*^ *=* .*03*.

**Fig 7:**
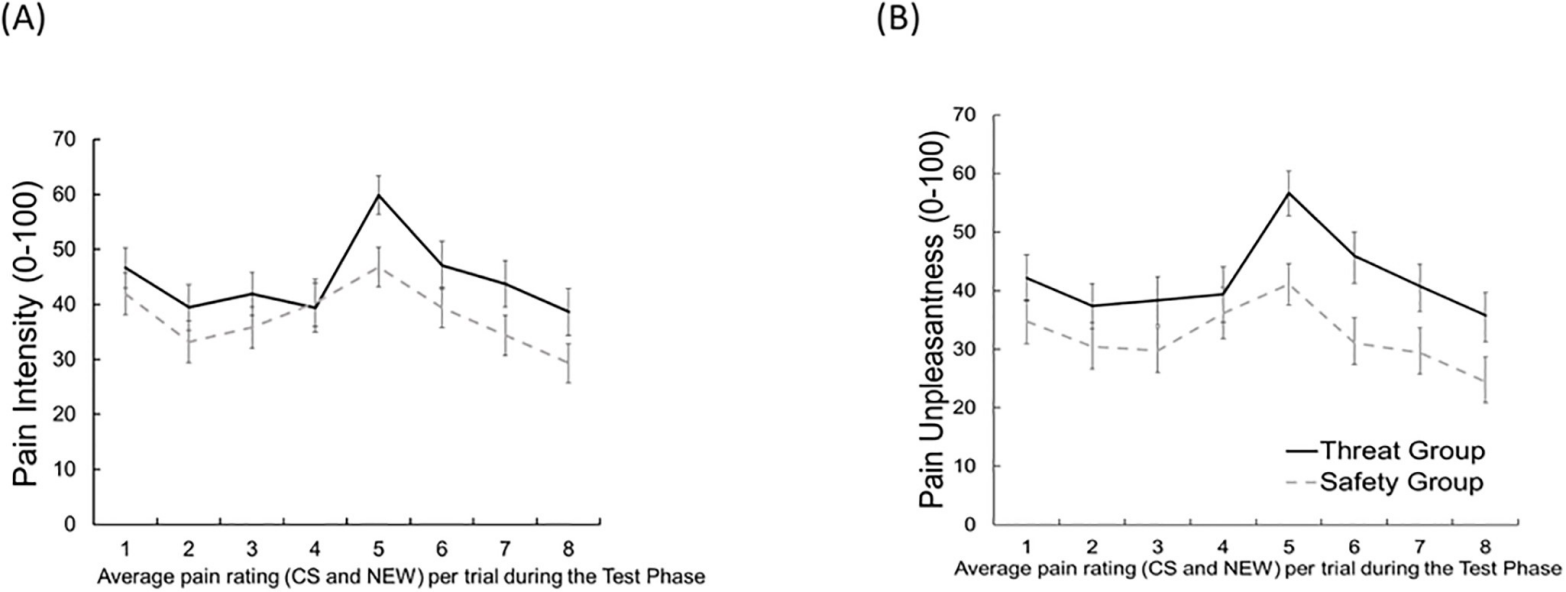
Pain intensity ratings and pain unpleasantness ratings averaged across cues. Mean (+SEM) (A) Pain Intensity Ratings and (B) Pain Unpleasantness Ratings, averaged across cues, separately for both groups, * *p* < .05. Asterisks indicate significant group difference at the specific trial.

*Exploratory analysis* point descriptively at higher ratings of both CS and NEW cues in the threat compared to the safety group at trial 5 (difference: 13.06, *t*(78) = 2.60, *p* = .01) directly after repositioning the thermode, and for trial 6 (difference: 7.69, *t*(78) = 1.35, *p* = .18), trial 7 (difference: 9.35, *t*(78) = 1.68, *p* = .09) and trial 8 (difference: 9.30, *t*(78) = 1.67, *p* = .09), irrespective of the presented cue (CS+/- or NEW). However, this effect became non-significant after accounting for the Bonferroni correction based on four tests and a corresponding *p*-value of *p* = 0.0125.(Fig 7). To get a better understanding of the temporal dynamics, we exploratory analyzed only the first trial. However, this did not reveal any effect for pain intensity.

For *pain unpleasantness* there was also no significant main effect of *group*, *F*(1, 78) = 3.20, *p* = .078, *ηp*^*2*^ *=* .04, nor of *cue*, *F*(1, 78) = .16, *p* = .69, *ηp*^2^ = .002. Similarly, analysis revealed no significant interaction of *cue* and *group*, *F*(1, 78) = 1.730, *p* = .192, *ηp*^*2*^ = .02, neither. Pain ratings differed over time for both groups, *F*(7, 546) = 2.20, *p* = .03, *ηp*^*2*^ = .03.

*Exploratory analyses* point at higher ratings of both kinds of stimuli in the threat compared to the safety group at trial 5 (difference: 15.54, *t*(78) = 2.68, *p* = .01), trial6 (difference: 14.89, *t*(78) = 2.49, *p* = .02), trial 7 (difference: 11.32, *t*(78) = 1.86, *p* = .07 and trial 8 (difference: 11.39, *t*(78) = 1.89, *p* = .06). However, this effect became non-significant after accounting for the Bonferroni correction based on eight tests and a corresponding *p*-value of *p* = 0.00625.(Fig 7). To get a better understanding of the temporal dynamics, we exploratory analyzed only the first trial. However, this did not reveal any effect for pain unpleasantness.

#### Heart rate

**Fig 8:**
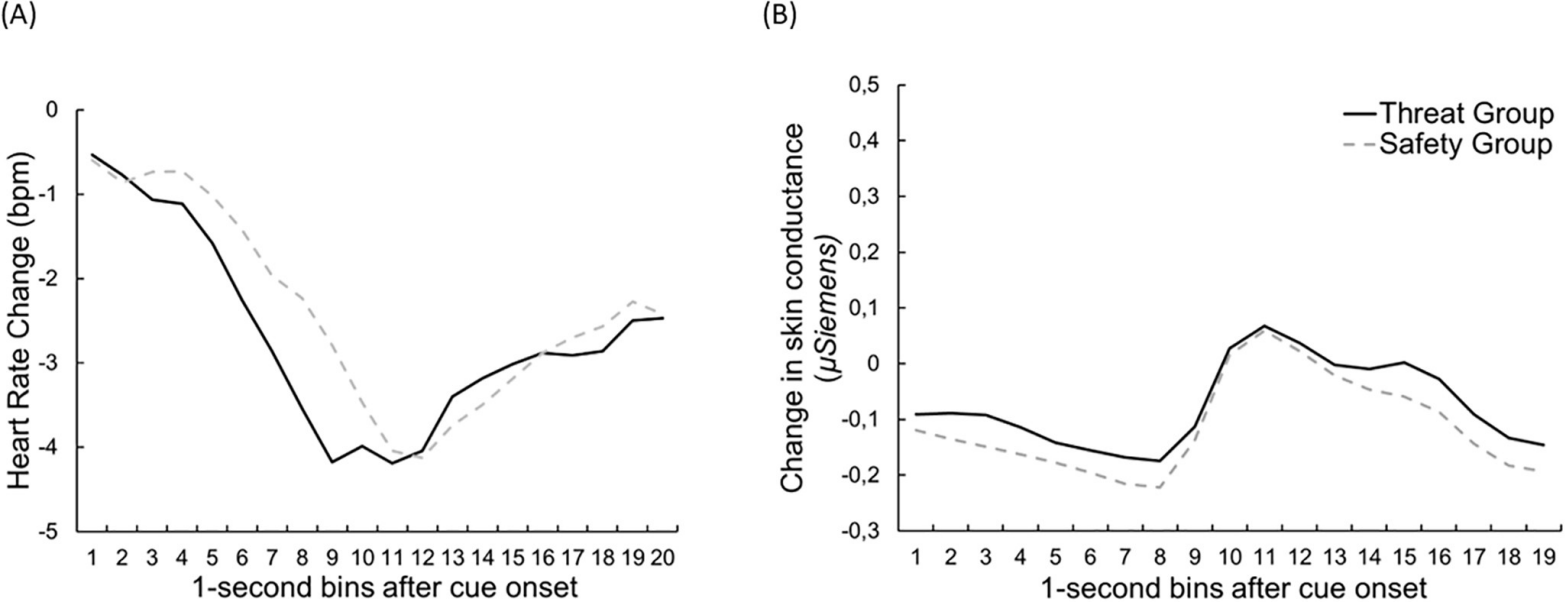
Time course of heart rate *and* of the change in skin conductance. Mean time course (1-s bins) of (A) Heart Rate (baseline-corrected 5 s before cue onset) for the averaged CS, separately for both experimental groups, during the Test Phase & (B) mean time course (1-s bins) of the change in Skin Conductance (baseline-corrected 5 s before cue onset) for the averaged CS+/- and CS.NEW during the Test Phase for the two experimental groups.

Analysis of heart rate revealed no main effect of *group* during the test phase (*F*(1, 57) = .86, *p* = .36, *ηp*^*2*^ = .02) nor of *cue* (*F*(1, 57) = .01, *p* = .91, *ηp*^*2*^ < .001. There was a significant main effect of *time*, *F*(19, 1083) = 17.24, *p* < .001, *ηp*^*2*^ = .23, *ε* = .18 indicating acceleration to the heat pain stimulus. There was no significant interaction of *cue* and *group*, *F*(1, 57) = 0.56, *p* = .46, ηp^2^ < .001, ε = .29, nor for *cue*, *time* and *group*, *F*(19, 1083) = 0.64, *p* = .06, *ηp*^2^ = .04, *ε* = .29. The mean time course for both groups is shown in Fig 8.

#### Skin conductance

**Fig 9:**
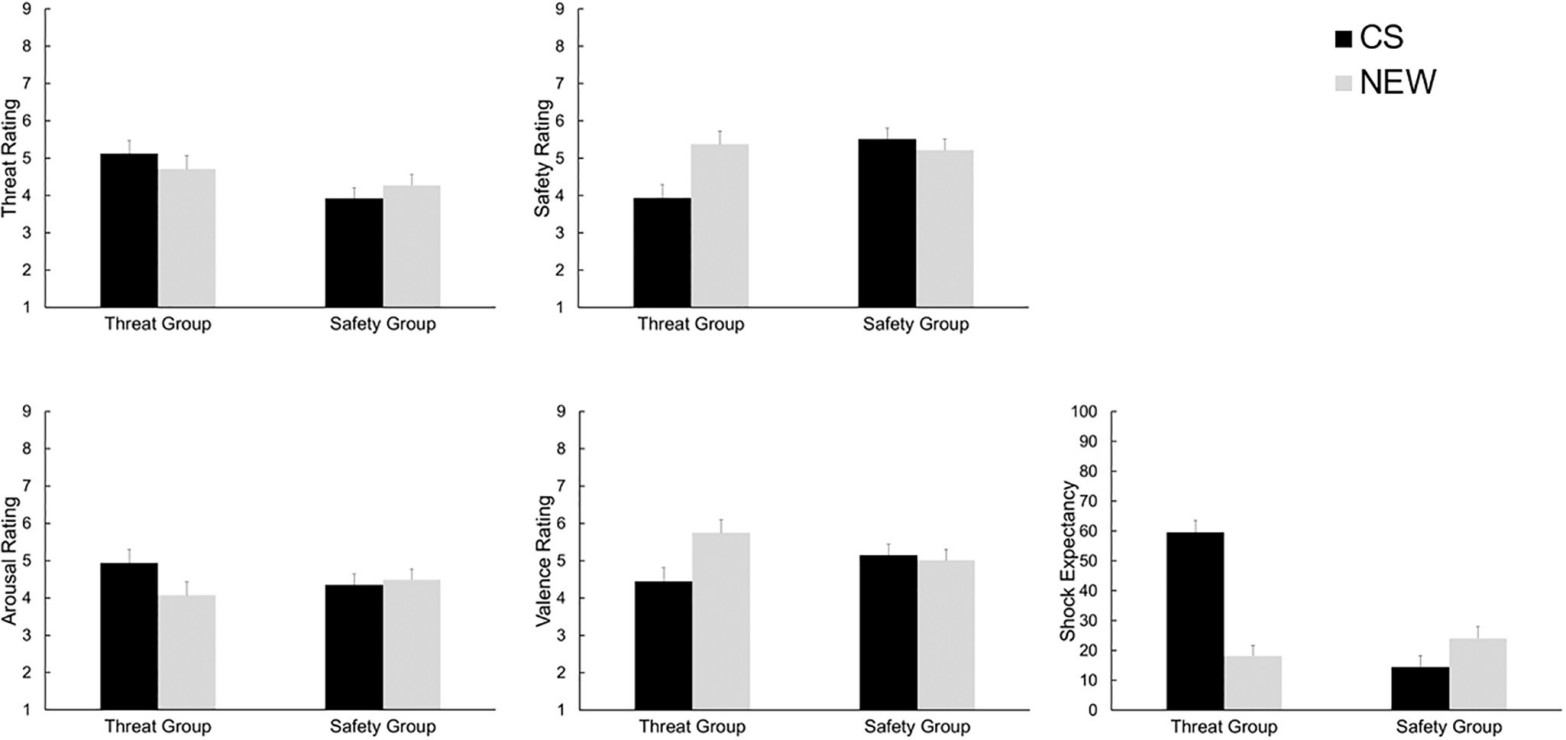
Affective cue ratings during the test phase. Mean (+ SEM) ratings for threat, safety, valence, arousal, and shock expectancy are depicted separately for both groups and cues, * p < .05.

Analysis revealed no main effect of *group*, *F*(1, 59) = .45, *p* = .51, *ηp*^2^ = .01. There was no difference between CS and NEW *cues*, *F*(1, 59) = 1.69, *p* = .20, *ηp*^2^ = .03. Similar as for HR a significant main effect of *time* was found, *F*(19, 1121) = 12.11, *p* < .001, *ηp*^*2*^ = .17, *ε* = .10, indicating a skin conductance reaction to the heat pain stimulus (Fig 9). Analysis of skin conductance level revealed no significant interaction of *cue* and *group*, *F*(1, 59) = .09, *p* = .76, *ηp*^2^ = .002. There were no further significant effects (interaction of *group* and t*ime*, *F*(19, 1121) = .15, *p* = .84, *ηp*^2^ = .002, *ε* = .10; interaction of *time* and *cue*, *F*(19, 1121) = 1.06, *p* = .39, *ηp*^2^ = .02, *ε* = .17; interaction of *time*, *group* and *cue*, *F*(19, 1121) = .76, *p* = .76, *ηp*^2^ = .01, *ε* = .10).

#### Cue ratings

*Cue ratings during the test phase*. Analysis of the *threat rating* during the test phase revealed no significant main effect of *group*, *F*(1, 78) = 3.38, *p* = .07, *ηp*^*2*^ = .04, neither was the main effect of *cue* significant, *F*(1, 78) = .02, *p* = .88, *ηp*^*2*^ < .001. There was no significant interaction of *cue* and *group*, *F*(1, 78) = 3.02, *p* = .09, *ηp*^*2*^ *=* .04 during the test phase.

Analysis of *safety rating* revealed no significant main effect of *group*, *F*(1, 78) = 2.19, *p* = .14, *ηp*^*2*^ = .03. There was a significant main effect of *cue*, *F*(1, 78) = 10.92, *p* < .001, *ηp*^*2*^ < .12, driven by higher safety ratings for the NEW cue compared to the CS. This was further qualified by a significant interaction of *cue* and *group*, *F*(1, 78) = 25.07, *p* < .001, *ηp*^*2*^ = .24, separate analysis for both groups revealed a significant main effect of *cue* in the threat group only, (*F*(1, 40) = 28.28, *p* < .001, *ηp*^*2*^ = .41) due to lower safety ratings for the CS compared to the NEW cue; in the safety group this comparison was not significant (*F*(1, 38) = 1.93, *p* = .17, *ηp*^*2*^ = .05).

*Arousal ratings* revealed no significant main effect of *group*, *F*(1, 78) = .06, *p* = .81, *ηp*^*2*^ = .001. There was no main effect of *cue*, *F*(1, 78) = 3.33, *p* = .07, *ηp*^*2*^ = .04, neither. There was a significant interaction of *cue* and *group*, *F*(1, 78) = 6.43, *p* = .01, *ηp*^*2*^ = .076. Separate analysis for both groups revealed a significant main effect of *cue* in the threat group (*F*(1, 40) = 6.95, *p* = .012, *ηp*^*2*^ = .15), but not in the safety group (*F*(1, 38) = 0.43, *p* = .52, *ηp*^*2*^ = .01).

Analysis of *valence ratings* revealed no significant main effect of *group*, *F*(1, 78) = .01, *p* = .91, *ηp*^*2*^*<* .001. But there was a significant main effect of *cue*, *F*(1, 78) = 8.95, *p* = .004, *ηp*^*2*^ < .10, the NEW cue was rated as more positive than the CS. This main effect was further qualified by a significant interaction of *cue* and *group*, *F*(1, 78) = 13.82, *p* < .001, *ηp*^*2*^ = .015. Separate analysis for both groups revealed more positive ratings of the NEW compared to the CS cue in the threat group only (*F*(1, 40) = 21.99, *p* < .001, *ηp*^*2*^ = .36), but not in the safety group (*F*(1, 38) = .27, *p* = .61, *ηp*^*2*^ = .01).

Analysis of *shock expectancy* during the test phase revealed a significant main effect of *cue*, *F*(1, 78) = 31.92, *p* < .001, *ηp*^*2*^ = .29, resulting from higher shock expectancy ratings for the CS compared to the NEW cue. A significant main effect of *group* indicated higher shock expectancy ratings of the threat group compared to the safety group, *F*(1, 78) = 15.80, *p* < .001, *ηp*^2^ = .17. This main effect was further qualified by a significant interaction of *cue* and *group*, *F*(1, 78) = 82.89, *p* < .001, *ηp*^*2*^ = .52. Separate analysis for both groups revealed significantly higher expectancy ratings for the CS compared to the NEW *cue* in the threat group, *F*(1, 40) = 87.56, *p* < .001, *ηp*^*2*^ = .69, and showed for the safety group significantly lower expectancy ratings for the CS compared to the NEW cue, *F*(1, 38) = 8.19, *p =* .01, *ηp*^*2*^ = .18.

Ratings of the cues in the test phase are presented in Fig 9.

*Exploratory analysis: Cue ratings of the CS(+/-) across acquisition and test phase*:

To map the time course of learning and extinction processes respectively, we analyzed the 4 cue ratings of the CS(+/-) during acquisition and test phase. The analysis demonstrates a clear difference between groups. Similar to the results of experiment 1 the ratings of the threat group across the experimental phases were relatively stable, while the safety extinction occurred not as fast as in experiment 1. Detailed analysis of the comparison of acquisition and test phase for the threat, safety, valence, arousal and shock expectancy ratings are presented in the supplement (S2 Fig).

## Discussion

### Pain modulation by threat and safety?

In the present studies we investigated the effect of a safety vs. threat manipulation—relative to a neutral reference condition (experiment 1)—on pain, and further, elaborated on the role of CS-US contingency, increase of variance of the ISI and verbal threat vs. safety instructions on the magnitude and stability of affect induction and related pain modulation (experiment 2). A couple of methodological changes were performed in experiment 2 relative to study 1, because of no or only weak modulation of pain through safety and threat respectively. In study 1 participants in the safety group might have associated the safety cue with the aversive electrical stimulus, even when the temporal separation of CS and US was strict. This potential association could have influenced the safety quality of the cue. However, neither the presentation of safety (CS-) nor threat (CS+) cues led to a significant modulation of pain ratings compared to the neutral condition (NEW), which was true for experiment 1 and 2. Similarly, physiological *pain responses* did not differ between groups or cues, except for SC responses during the test phase of experiment 1, where SC was increased for the CS (+ and -) compared to the NEW cue. However, SC and HR *cue responses* during the test phase of experiment 1 and 2, revealed no differences between groups and CS conditions. Affective cue ratings during the test phase revealed that only in the threat group, the CS+ was rated as more aversive than the NEW cue (in experiment 1 safety, valence, and shock expectancy ratings and in experiment 2 additionally valence ratings), while in the safety group, there were no significant differences between the CS- and the NEW cue.

*Manipulation check and design adaptation in study 2*:

Cue ratings of the CS during acquisition of experiment 1 indicate a more long-winded induction of safety than of threat, but especially for experiment 2 differences between the safety and threat group, as expected. The comparison of acquisition and test phase regarding ratings of the CS+ and CS—demonstrates relatively stable ratings in the threat group across the experimental phases, while analysis of the ratings of the safety group suggest rapid safety extinction, very likely resulting from a newly build association of the CS- and the heat pain stimulation, challenging or even overwriting its previously established role as a safety signal.

In line with findings demonstrating the crucial role of verbal instructions regarding the induction of threat and safety in the context of (instructed) conditioning paradigms [2, 3, 27, 42], we provided additional verbal instructions explicating the role of the CS-/+ in experiment 2. Indeed, the methodological changes in experiment 2 led to a more pronounced differentiation between the different cues, especially regarding shock expectancy ratings. However, learned safety remained rather unstable with safety still extinguishing rapidly. We did not find an increase of pain following the CS+ presentation in the threat group, which might be due to the comparison relative to a newly introduced neutral cue during the test phase, instead of a previously conditioned CS-, as performed in earlier studies [2]. The comparison of CS+ and NEW affective cue ratings revealed a significant effect between conditions for safety, valence and especially for shock expectancy ratings, but not for the threat and arousal ratings. The differentiation between CS+ and NEW cue might have been insufficient to result in a significant emotional modulation of pain compared to previous findings [2]. The same was true for the safety group, here the comparison of CS- and NEW cue during the test phase revealed only very small differences. Separate analysis revealed only for the threat group significant differences for the safety and valence ratings, instead in the safety group, no significant difference between CS- and NEW cue was found, thus making the modulation of pain by safety less likely. Admittedly, the long ISI in our design may be challenging for participants with regard to attentional resources [43] interfering with the learning experience and related affect induction, nevertheless, especially for safety conditioning, long ISI seem indispensable, as trace conditioning was found to take place even when the US follows the CS up to 10s later [28]. Similarly, in case the US precedes the (next) CS presentation for less than 10s, backward conditioning may be initiated, converting the CS to a threat signal [26]. We cannot rule out that participants of the safety group, despite strict temporal separation of CS and US, perceived the safety cue to some degree as threating. Although, shock expectancy ratings revealed a significant difference between the CS- and CS+ between groups, and further receiving a shock was rated as less probable following the CS- compared to the NEW cue. Still ratings of the CS- in the safety group were on average about 15 per 100, which at least speaks for some association between the safety cue and shock.

*Interactions of Pain and Emotion Processing*—*Implications for future research*:

Future research on safety per se and pain modulation by safety inductions, should systematically explore temporal characteristics of CS and US presentation, or even consider the implementation of experimental safety manipulations, such as discriminative paradigms (AX-/BX+), which might establish a safety association that is more robust against extinction effects [44, 45]. Future research should also focus on differentiating the underlying mechanisms, such as extinction of safety and new or competing threat learning following heat pain administrations. Considering the rapid extinction process we observed, future studies should account for the temporal dynamics of learning processes.

In the present studies we deliberately decided against a differential conditioning paradigm and chose an explicit unpaired procedure, because of the unique possibility to investigate the influence of a safety cue in the absence of a threat cue, which otherwise promotes the direct comparison between groups or cues, leading to a CS evaluation and thus emotional status of safety or threat, which relies on the comparison to an orthogonal condition. Furthermore, the explicit unpaired procedure has been already used repeatedly in animal studies to investigate safety [16, 21–23] and Pollak et al. [21] already successfully transferred the paradigm into human research, showing that an unpaired conditioned stimulus is able to acquire the quality of a safety signal. However, one crucial deviation in our design from the study by Pollak et al. was the administration of heat pain stimuli during the test phase. As already outlined above, this might have motivated a new learning process competing with the previously acquired CS association resulting from the acquisition phase, leading to an *extinction of safety* [26, 46]. Although the CS- still was never paired with the electrical shock, it was now reliably accompanied by heat pain at a 100% contingency.

While the effect of emotion on pain is well-documented, such that in accordance with the concept of motivational priming the (in)congruence of an affective stimulus with the sensation of concurrent pain may lead to a pain facilitating or reducing effect, respectively [1, 6, 7, 47], the impact of pain serving as a motivational context and by that changing the processing of emotions or interfering with learning processes, was investigated way less frequently. For instance, Meulders [48] argues, that the experience of pain may act as a prime for precautious behavior, which facilitates defensive responses to keep further costs and damage low. In this context, Godinho, Magnin [49] found that the administration of pain led to a reduction of positive affective responses following pleasant picture presentation. Similarly, it was found that concurrently administered pain led to more negative valence and higher arousal ratings of affective face stimuli, which at the same time resulted in increased pain ratings [9]. What is more, the administration of tonic painful stimuli was shown to result in a general decrease of early neurophysiological correlates of affective face processing [50], demonstrating high demands of pain for attention, interfering with concurrent cognitive and affective processes [51].

Considering these findings, the present results indicate that the administration of painful heat, not only led to a newly build threat association, but especially hampered the consolidation of the CS-, given the motivational incongruence of pain and safety and the high saliency of pain, leading to cognitive interference. It was demonstrated that stress, resulting from hand immersion into painfully cold water, led to reduced threat extinction, suggesting a long lasting effect of pain on learning, since the painful stress experience anteceded threat acquisition by days [52]. Similarly, chronic pain patients compared to healthy controls demonstrated reduced differential learning and elevated threat generalization in threat conditioning paradigms [53]. In addition to aberrant threat learning and extinction, deficient safety learning was demonstrated for chronic pain patients and patients with anxiety disorders [44, 48, 54].

In both experiments pain ratings for the threat group were higher in the second half of the experiment compared to the safety group. In experiment 2, the increase after repositioning the thermode was especially pronounced and prolonged in the threat group. With regard to motivational priming applied to the context of pain [19, 20], for participants of the threat group the acutely increased pain sensation in later trials might have served as a salient somatosensory reminder of the aversive conditioning procedure, inducing negative affect and increased pain.

## Conclusion

The present data revealed no modulation by safety–following safety induction using an adapted explicit unpaired procedure established by Pollak [21]. Instead, both studies demonstrate the challenge to establish and especially maintain safety in the context of concurrently experienced pain. Future studies are necessary, which address the time course of threat and especially safety extinction—in the context of pain—elucidating the role of hampered safety learning (and rapid safety extinction) for pain processing and the devolvement of chronic pain and its comorbidities.

## Supporting information

S1 Fig Affective cue ratings of the CS during acquisition and test phase for both groups. (TIF)

S2 Fig Affective cue ratings of the CS during acquisition and test phase for both groups. (TIF)

S1 File Experiment 1. Additional information of participants. Additional Information of Participants Before the main experiment participants completed the state version of the *State-Trait Anxiety Inventory*, STAI-S [55, 56], the *Positive and Negative Affect Schedule*, PANAS [57], the *Pain Sensitivity Questionnaire*, PSQ [58], the *Pain Catastrophizing Scale*, PCS [59, 60]. After the experiment the following questionnaires were assessed: the *Resilience Scale*, RS-25 [61], the trait version of the *State-Trait Anxiety Inventory*, STAI-T [55, 56], the *Life-Orientation-Test Revised*, LOT-R [62, 63], the *Expressions of Spirituality*, ASP2.1 [64, 65], the *Beck Depression-Inventory*, BDI-II [66, 67], *the Sensitivity to Punishment and Sensitivity to Reward*, SPSRQ [68], the *Experience in close relationships- revised* ECR-RD [69, 70] and the *Anxiety Sensitivity Index-3*, ASI3 [71] was assessed. In general, the two groups did not differ in their test scores (Table 1) apart from their PSQ (*p* = .03), where the threat group had higher scores and the subscale of sensitivity to punishment scale of the SPSRQ (*p* = .02), where the safety group had higher scores. The mean questionnaire scores and standard deviations are shown in S1 Table.
(DOCX)

S2 File Experiment 2. Additional information of participants. The assessed questionnaires in experiment 2 after the experiment were reduced in comparison to experiment 1 to keep the total time limited, therefore only the following questionnaires were assessed: the *Resilience Scale*, RS-25 [61], the trait version of the *State-Trait Anxiety Inventory*, STAI-T [55, 56], the *Life-Orientation-Test Revised*, LOT-R [62, 63], the *Expressions of Spirituality*, ASP2.1 [64, 65]. The *Beck Depression-Inventory*, BDI-II [66, 67], *the Sensitivity to Punishment and Sensitivity to Reward*, SPSRQ [68], the *Experience in close relationships- revised* ECR-RD [69, 70] and the *Anxiety Sensitivity Index-3*, ASI3 [71] were no longer assessed. The two groups did not differ in their test scores (S2 Table).
(DOCX)

S1 Table Mean scores of pain threshold and questionnaires in the two experimental groups. (DOCX)

S2 Table Mean scores in pain threshold and questionnaires in the two experimental groups. (DOCX)
