# Supplementary figures and images for: Better safe than sorry?—On the influence of learned safety on pain perception
Source: PLoS One. 2023 Nov 7;18(11):e0289047. doi: 10.1371/journal.pone.0289047 (PMC10629634; doi:10.1371/journal.pone.0289047)

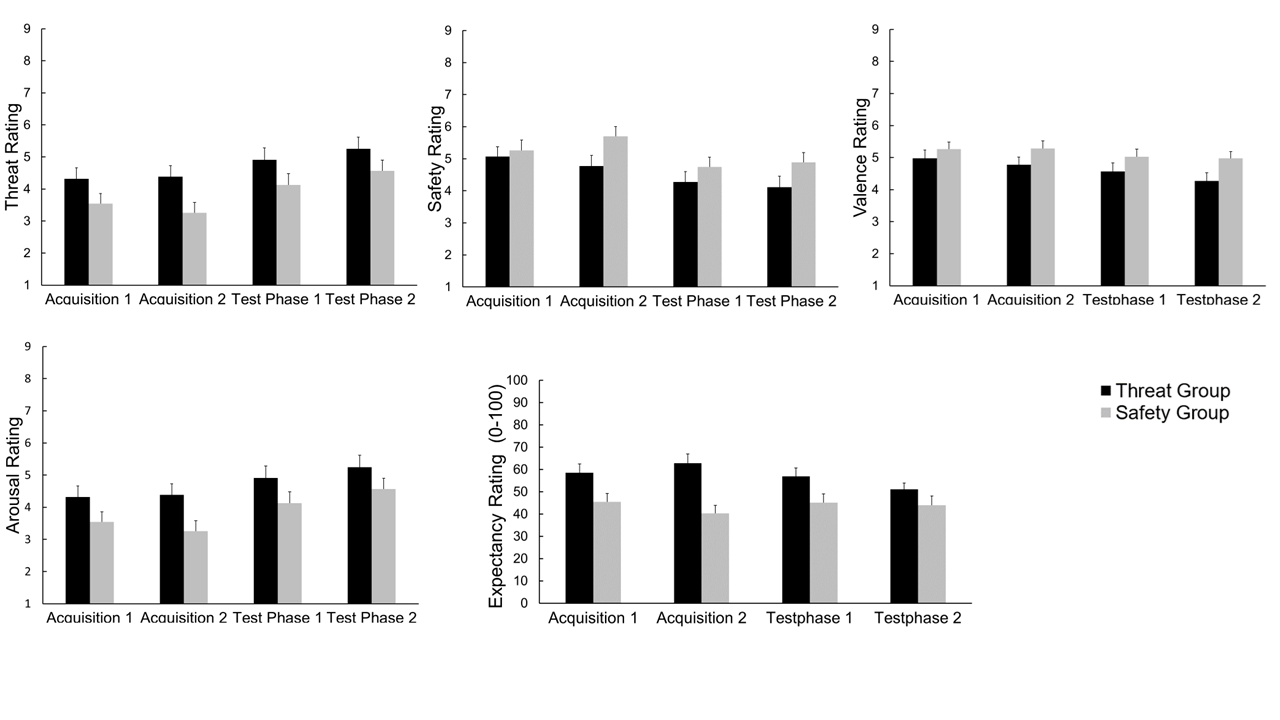

Supplement: S1 Fig — (TIF) [file pone.0289047.s001.tif]

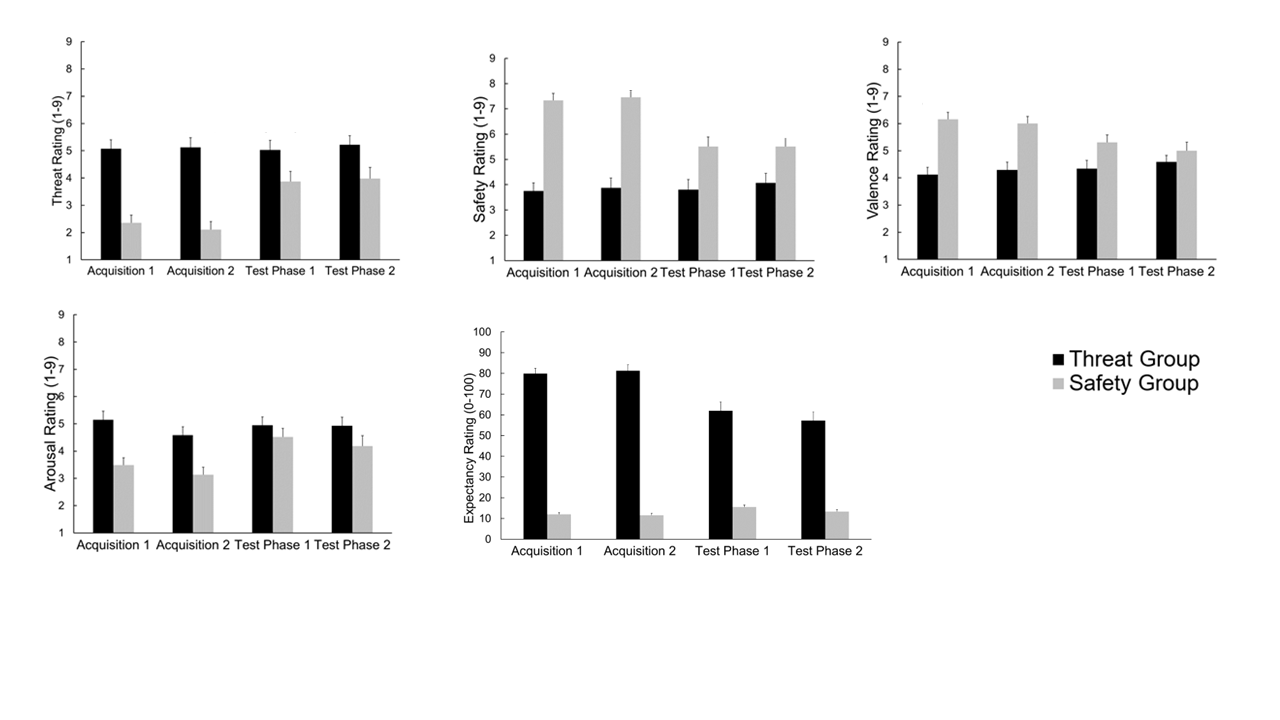

Supplement: S2 Fig — (TIF) [file pone.0289047.s002.tif]
