# Supplementary material for: Better safe than sorry?—On the influence of learned safety on pain perception
Source: PLoS One. 2023 Nov 7;18(11):e0289047. doi: 10.1371/journal.pone.0289047 (PMC10629634; doi:10.1371/journal.pone.0289047)
Supplement: S1 File — Additional information of participants. Additional Information of Participants Before the main experiment participants completed the state version of the State-Trait Anxiety Inventory, STAI-S [55, 56], the Positive and Negative Affect Schedule, PANAS [57], the Pain Sensitivity Questionnaire, PSQ [58], the Pain Catastrophizing Scale, PCS [59, 60]. After the experiment the following questionnaires were assessed: the Resilience Scale, RS-25 [61], the trait version of the State-Trait Anxiety Inventory, STAI-T [55, 56], the Life-Orientation-Test Revised, LOT-R [62, 63], the Expressions of Spirituality, ASP2.1 [64, 65], the Beck Depression-Inventory, BDI-II [66, 67], the Sensitivity to Punishment and Sensitivity to Reward, SPSRQ [68], the Experience in close relationships- revised ECR-RD [69, 70] and the Anxiety Sensitivity Index-3, ASI3 [71] was assessed. In general, the two groups did not differ in their test scores (Table 1) apart from their PSQ (p = .03), where the threat group had higher scores and the subscale of sensitivity to punishment scale of the SPSRQ (p = .02), where the safety group had higher scores. The mean questionnaire scores and standard deviations are shown in S1 Table. (DOCX) [file pone.0289047.s003.docx]

S1 Experiment 1

Additional Information of Participants

Before the main experiment participants completed the state version of the *State-Trait Anxiety Inventory*, STAI-S [59, 60], the *Positive and Negative Affect Schedule*, PANAS [61], the *Pain Sensitivity Questionnaire,* PSQ [62], the *Pain Catastrophizing Scale,* PCS [63, 64]. After the experiment the following questionnaires were assessed: the *Resilience Scale*, RS-25 [65], the trait version of the *State-Trait Anxiety Inventory,* STAI-T [59, 60], the *Life-Orientation-Test Revised*, LOT-R [66, 67], the *Expressions of Spirituality*, ASP2.1 [68, 69], the *Beck Depression-Inventory*, BDI-II [70, 71], *the Sensitivity to Punishment and Sensitivity to Reward*, SPSRQ [72], the *Experience in close relationships- revised* ECR-RD [73, 74] and the *Anxiety Sensitivity Index-3*, ASI3 [75] was assessed. In general, the two groups did not differ in their test scores (Table 1) apart from their PSQ (*p* = .03), where the threat group had higher scores and the subscale of sensitivity to punishment scale of the SPSRQ (*p* = .02), where the safety group had higher scores. The mean questionnaire scores and standard deviations are shown in Table S1.

*Cue ratings of the CS(+/-) across acquisition and test phase:* To map the time course of learning and extinction processes respectively, we additionally analyzed the 4 cue ratings of the CS(+/-) during acquisition and test phase.

Analysis of *threat ratings* revealed a significant main effect of *group*, due to higher ratings of the threat group compared to the safety group, *F*(1, 88) =3.98, *p* = .049*, ηp²* = .04. There was also a significant main effect of *time*, *F*(3, 264) = 13.64, *p* < .001, *ηp² =* .13, ε = .76, indicating that the CS (+ and -) became more threatening over the time course of the experiment. The interaction of *time* and *group*, *F*(3, 264) = 0.50, *p* = .69, *ηp² =* .01 was not significant.

Analysis of *safety rating* revealed no main effect of *group*, *F*(1, 88) = 1.68, *p* = .20*, ηp²* = .02. There was a significant main effect of *time*, *F*(3, 264) = 9.61, *p* < .001, *ηp² =* .09, ε = .84, indicating that the CS was rated as less safe in the advanced stage of the experiment. These main effects were further qualified by a significant interaction of *time* and *group*, *F*(3, 264) = 1.81, *p* = .15, *ηp² =* .02. Separate analysis for each block revealed a significant group difference after the second acquisition block, *t*(88) = -2.05, *p* = .04). The other safety ratings did not differ between groups (acquisition 1: *t*(88) = -.43, *p* = .67), test phase 1: *t*(88) = -1.03, *p* = .31), test phase 2: *t*(88) = -1.69, *p* = .09)).

Analysis of *arousal rating* revealed no main effect of *group*, *F*(1, 88) = 2.77, *p* = .10*, ηp²* = .03. There was a significant increase of arousal over *time*, *F*(3, 264) = 7.30, *p* < .001, *ηp² =* .08, ε = .78. Also the interaction of *time* and *group* was significant, *F*(3, 264) = 3.03, *p* = .03, *ηp² =* .03: Separate analysis for both groups revealed only a significant main effect of *time* for the safety group (*F*(3, 135) = 8.14, *p* < .001, *ηp² =* .15, ε = .66), but not for the threat group (*F*(3, 129) = .95, *p* = .42, *ηp² =* .02, ε = .84), indicating that the lower arousal ratings of the safety group during the acquisition, increased within the test phase.

Analysis of *valence ratings* revealed no main effect of *group*, *F*(1, 88) = 2.71, *p* = .10*, ηp²* = .03., but a significant main effect of *time*, *F*(3, 264) = 5.02, *p* = .01, *ηp² =* .05, ε = .72, indicating that the cues were rated as less positive over the time course of the experiment. There was no significant interaction of *time* and *group*, *F*(3, 264) = 0.76, *p* = .52, *ηp² =* .01.

The analysis of *shock expectancy ratings* revealed higher ratings for the threat compared to the safety group, *F*(1, 88) =9.06, *p* = .003*, ηp²* = .09. There was no significant main effect of *time* (*F*(3, 264) = 1.20, *p* = .31, *ηp² =* .01, ε = .72). Further, the interaction of *time* and *group* was significant, *F*(3, 264) = 2.93, *p* = .03, *ηp² =* .03 for the shock expectancy ratings. Separate analysis for both groups revealed a decrease of shock expectancy over time for the threat group only (*F*(3, 129) = 4.22, *p* = .001, *ηp² =* .089), but not for the safety group (*F*(3, 135) = .64, *p* = .59, *ηp² =* .01).
