# Supplementary material for: Better safe than sorry?—On the influence of learned safety on pain perception
Source: PLoS One. 2023 Nov 7;18(11):e0289047. doi: 10.1371/journal.pone.0289047 (PMC10629634; doi:10.1371/journal.pone.0289047)
Supplement: S2 File — Additional information of participants. The assessed questionnaires in experiment 2 after the experiment were reduced in comparison to experiment 1 to keep the total time limited, therefore only the following questionnaires were assessed: the Resilience Scale, RS-25 [61], the trait version of the State-Trait Anxiety Inventory, STAI-T [55, 56], the Life-Orientation-Test Revised, LOT-R [62, 63], the Expressions of Spirituality, ASP2.1 [64, 65]. The Beck Depression-Inventory, BDI-II [66, 67], the Sensitivity to Punishment and Sensitivity to Reward, SPSRQ [68], the Experience in close relationships- revised ECR-RD [69, 70] and the Anxiety Sensitivity Index-3, ASI3 [71] were no longer assessed. The two groups did not differ in their test scores (S2 Table). (DOCX) [file pone.0289047.s004.docx]

**S2 Experiment 2**

Additional Information of Participants

The assessed questionnaires in experiment 2 after the experiment were reduced in comparison to experiment 1 to keep the total time limited, therefore only the following questionnaires were assessed: the *Resilience Scale*, RS-25 [65], the trait version of the *State-Trait Anxiety Inventory,* STAI-T [59, 60], the *Life-Orientation-Test Revised*, LOT-R [66, 67], the *Expressions of Spirituality*, ASP2.1 [68, 69].

The *Beck Depression-Inventory*, BDI-II [70, 71], *the Sensitivity to Punishment and Sensitivity to Reward*, SPSRQ [72], the *Experience in close relationships- revised* ECR-RD [73, 74] and the *Anxiety Sensitivity Index-3*, ASI3 [75] were no longer assessed. The two groups did not differ in their test scores (Table S2).

*Cue ratings of the CS(+/-) across acquisition and test phase:*

To map the time course of learning and extinction processes respectively, we analyzed the 4 cue ratings of the CS(+/-) during acquisition and test phase. Analysis of *threat rating* revealed a significant main effect of *group*, *F*(1, 78) = 25.03, *p* < .001, *ηp² = .24*) and a significant main effect of *time*, *F*(3, 234) = 10.31, *p* < .001, *ηp² = .12,*  ε = .59). There was a significant interaction of *time* and *group*, *F*(3, 234) = 9.69, *p* < .001, *ηp² = .11.* Separate analysis for both groups only revealed a significant main effect of *time* in the safety group (*F*(3, 114) =13.84, *p* < .001*, ηp²* = .27, ε = .46), but not for the threat group (*F*(3, 120) = .24, *p* = .87*, ηp²* = .01), indicating that the ratings of the threat group were quite stable throughout the experiment, whereas threat ratings of the safety group decreased from acquisition to test phase.

Analysis of *safety rating* revealed a significant main effect of *group*, *F*(1, 78) = 33.55, *p* < .001, *ηp² = .30*) and a significant main effect of *time*, *F*(3, 234) = 13.80, *p* < .001, *ηp² = .15,* ε = .62). There was also a significant interaction of *time* and *group*, *F*(3, 234) = 17.67, *p* < .001, *ηp² = .19*. Separate analysis for both groups only revealed a significant main effect of *time* in the safety group (*F*(3, 114) =20.42, *p* < .001*, ηp²* = .35, ε = .55), but not for the threat group (*F*(3, 120) = .96, *p* = .39*, ηp²* = .02, ε = .74), indicating that the ratings of the threat group were quite stable through the experiment, whereas the safety ratings of the safety group decreased significantly from acquisition 2 to test phase 1 (*t*(38) = 4,77, *p* < .001).

Analysis of *arousal ratings* also revealed a main effect of *group, F*(1, 78) = 8.49, *p* = .01, *ηp² = .*10), because of higher ratings of the threat group and there was a significant main effect of *time, F*(3, 234) = 7.23, *p* < .001, *ηp² = .09,* ε = .62). There was also a significant interaction of *time* and *group*, *F*(3, 234) = 4.20, *p* = .006, *ηp²* = .05. Separate analysis for both groups only revealed a significant main effect of *time* in the safety group (*F*(3, 114) = 7.18, *p* = .004*, ηp²* = .16, ε = .51), but not for the threat group (*F*(3, 120) = 2.16, *p* = .10*, ηp²* = .05), indicating that the ratings of the threat group were quite stable through the experiment, whereas the arousal ratings of the safety group rose during the test phase.

Analysis of *valence ratings* revealed a main effect of *group, F*(1, 78) = 14.73, *p* < .001, *ηp² = .16*), because of higher ratings of the safety group. There was no significant main effect of *time, F*(3, 234) = 2.57, *p* = .07, *ηp² = .03,* ε = .82).There was a significant interaction of *time* and *group*, *F*(3, 234) = 9.15, *p* < .001, *ηp²* = .11*.* Separate analysis for both groups only revealed a significant main effect of *time* in the safety group (*F*(3, 114) =17.17, *p* = .001*, ηp²* = .17, ε = 69), but not for the threat group (*F*(3, 120) = 1.81, *p* = .15*, ηp²* = .04, ε = .93), indicating that the ratings of the threat group were quite stable through the experiment, whereas as time goes by the cues were rated as less positive by the safety group.

The threat group had higher *shock expectancy ratings* compared to the safety group, *F*(1, 78) =179.06, *p* < .001*, ηp²* = .69. There was a main effect of *time*, *F*(3, 234) = 8.77, *p* < .001, *ηp² = .10,* ε = .63. Analysis also revealed a significant interaction of *time* and *group*, *F*(3, 234) = 13.63, *p* > .001, *ηp² =* .15, for the shock expectancy ratings. Separate analysis for both groups only revealed a decrease of shock expectancy over time for the threat group (*F*(3, 120) = 20.87, *p* < .001, *ηp² =* .34), but not for the safety group (*F*(3, 114) = .49, *p* = .69, *ηp² =* .01).

To summarize, the analysis of all affective cue and expectancy ratings showed that the threat group remains relatively stable in their ratings from acquisition to the test phase, whereas in safety group, evaluation of the CS- changes significantly, becoming more like the CS+ over time, likely due to the positive association of cue and pain presentation.
