# Supplementary material for: Better safe than sorry?—On the influence of learned safety on pain perception
Source: PLoS One. 2023 Nov 7;18(11):e0289047. doi: 10.1371/journal.pone.0289047 (PMC10629634; doi:10.1371/journal.pone.0289047)
Supplement: S1 Table — (DOCX) [file pone.0289047.s005.docx]

**S1 Table**. **Mean scores of pain threshold and questionnaires in the two experimental groups**

| *Measure* | *safety group*  *(n = 46)* | | *threat group*  *(n = 44)* | |  | |  | |  |
| --- | --- | --- | --- | --- | --- | --- | --- | --- | --- |
|  | M | SD | M | SD | | t | | P | |
| Age | 23.63 | 3.65 | 25.80 | 4.061 | | 2.66 | | **.01*** | |
| Heat pain threshold (°C) | 43.109 | 1.66 | 43.068 | 2.60 | | 0.09 | | .93 | |
| Electric pain threshold (mA) | 0.745 | 0.54 | 0.873 | 0.576 | | 1.09 | | .28 | |
| STAI_State | 33.87 | 4.81 | 36.23 | 7.72 | | 1.73 | | .09 | |
| PANAS_Positive | 28.63 | 5.96 | 29.86 | 5.92 | | .99 | | .33 | |
| PANAS_Negative | 11.83 | 1.68 | 12.77 | 4.33 | | 1.36 | | .18 | |
| PSQ_Total | 4.16 | 1.36 | 4.79 | 1.42 | | 2.16 | | **.03*** | |
| PCS | 15.76 | 9.45 | 16.89 | 9.54 | | .56 | | .58 | |
| RS25 | 139.24 | 23.59 | 135.09 | 20.15 | | .25 | | .81 | |
| STAI_TRAIT | 37.37 | 8.58 | 37.73 | 10.12 | | .18 | | .86 | |
| LOT_R | 17,15 | 3.94 | 16.23 | 5.13 | | .96 | | .34 | |
| ASP_ religious orientation | 31.22 | 28.26 | 26.39 | 24.78 | | .86 | | .39 | |
| ASP_search for insight | 58.07 | 23.71 | 55.37 | 21.17 | | .57 | | .57 | |
| ASP-conscious interaction | 76.30 | 16.11 | 73.75 | 15.67 | | .76 | | .45 | |
| ASP_transcendence-conviction | 48.37 | 27.49 | 41.71 | 23.84 | | 1.23 | | .22 | |
| BDI-II | 7.02 | 6.45 | 7.45 | 6.45 | | .33 | | .74 | |
| SPSRQ_punish | 11.50 | 5.02 | 8.91 | 5.24 | | 2.40 | | .02* | |
| SPSRQ_reward | 10.59 | 3.82 | 11.48 | 4.03 | | 1.08 | | .29 | |
| ECR_RD_Bang | 50.76 | 19.16 | 52.45 | 20.97 | | .40 | | .69 | |
| ECR_RD_Bver | 42.15 | 16.27 | 44.05 | 18.96 | | .51 | | .61 | |
| ASI3 | 19.22 | 10.14 | 15.50 | 9.27 | | 1.81 | | .07 | |

STAI_State/ Trait. State/Trait Anxiety Inventory; PANAS, Positive Affect/Negative Affect Schedule; PSQ, Pain Sensitivity Questionnaire; PCS, Pain Catastrophizing Scale Questionnaire; RS25, Resilience Scale; LOT_R, Life-Orientation-Test; ASP, Aspects of Spirituality, religious orientation, search for insight, conscious interaction, transcendence-conviction; BDI-II, Beck Depression-Inventory; SPSRQ, Sensitivity to Punishment and Sensitivity to Reward; ECR_RD, Experience in close relationships- revised, attachment-related avoidance and attachment-related anxiety; ASI3, Anxiety Sensitivity Index-3; * = *p* <.05
