# Supplementary material for: Better safe than sorry?—On the influence of learned safety on pain perception
Source: PLoS One. 2023 Nov 7;18(11):e0289047. doi: 10.1371/journal.pone.0289047 (PMC10629634; doi:10.1371/journal.pone.0289047)
Supplement: S2 Table — (DOCX) [file pone.0289047.s006.docx]

**Table S2**. **Mean scores in pain threshold and questionnaires in the two experimental groups**

| *Measure* | *safety group*  *(n = 39)* | | *threat group*  *(n = 41)* | |  |  |
| --- | --- | --- | --- | --- | --- | --- |
|  | M | SD | M | SD | t | p |
| Age | 25.08 | 5.03 | 24.39 | 3.68 | -0.69 | .49 |
| Heat pain threshold (°C) | 42.74 | 2.20 | 43.21 | 2.23 | 0.93 | 0.35 |
| Electrical pain threshold (mA) | 1.05 | 0.69 | 1.02 | 0.87 | -0.19 | 0.85 |
| STAI_State | 33.38 | 7.42 | 34.95 | 5.72 | 1.06 | .29 |
| PANAS_Positive | 31.56 | 7.79 | 29.48 | 5.98 | -1.34 | 0.18 |
| PANAS_Negative | 12.46 | 3.42 | 11.68 | 1.97 | -1.24 | 0.22 |
| PSQ_Total | 3.80 | 1.00 | 3.37 | 1.23 | -1.71 | 0.09 |
| PCS | 16.05 | 7.62 | 14.66 | 8.03 | -0.79 | 0.43 |
| RS25 | 132.64 | 19.31 | 132.88 | 17.05 | 0.06 | 0.95 |
| STAI_TRAIT | 37.92 | 9.97 | 37.54 | 9.21 | -0.18 | .86 |
| LOT_R | 17.28 | 3.83 | 16.10 | 3.83 | -1.39 | 0.17 |
| ASP_ religious orientation | 40.31 | 26.58 | 31.57 | 28.56 | -1.42 | 0.16 |
| ASP_search for insight | 67.22 | 21.08 | 58.89 | 20.70 | -1.78 | 0.08 |
| ASP-conscious interaction | 82.95 | 34.25 | 74.51 | 13.73 | -1.45 | 0.15 |
| ASP_transcendence-conviction | 52.40 | 25.15 | 49.09 | 31.02 | -0.52 | 0.60 |

STAI_State/ Trait, State/Trait Anxiety Inventory; PANAS, Positive Affect/Negative Affect Schedule; PSQ, Pain Sensitivity Questionnaire; PCS, Pain Catastrophizing Scale Questionnaire; RS25, Resilience Scale; LOT_R, Life-Orientation-Test; ASP, Aspects of Spirituality, religious orientation, search for insight, conscious interaction, transcendence-conviction, * = *p* <.05.
